# Multi-Omic Analyses Reveal Habitat Adaptation of Marine Cyanobacterium *Synechocystis* sp. PCC 7338

**DOI:** 10.3389/fmicb.2021.667450

**Published:** 2021-05-13

**Authors:** Yujin Jeong, Seong-Joo Hong, Sang-Hyeok Cho, Seonghoon Yoon, Hookeun Lee, Hyung-Kyoon Choi, Dong-Myung Kim, Choul-Gyun Lee, Suhyung Cho, Byung-Kwan Cho

**Affiliations:** ^1^Department of Biological Sciences, Korea Advanced Institute of Science and Technology, Daejeon, South Korea; ^2^Department of Biological Engineering, Inha University, Incheon, South Korea; ^3^Department of Biological Sciences and Bioengineering, Inha University, Incheon, South Korea; ^4^Institute of Pharmaceutical Research, College of Pharmacy, Gachon University, Incheon, South Korea; ^5^College of Pharmacy, Chung-Ang University, Seoul, South Korea; ^6^Department of Chemical Engineering and Applied Chemistry, Chungnam National University, Daejeon, South Korea; ^7^Innovative Biomaterials Center, KI for the BioCentury, Korea Advanced Institute of Science and Technology, Daejeon, South Korea; ^8^Intelligent Synthetic Biology Center, Daejeon, South Korea

**Keywords:** cyanobacteria, marine *Synechocystis* sp., photosynthesis, genome, transcriptome

## Abstract

Cyanobacteria are considered as promising microbial cell factories producing a wide array of bio-products. Among them, *Synechocystis* sp. PCC 7338 has the advantage of growing in seawater, rather than requiring arable land or freshwater. Nonetheless, how this marine cyanobacterium grows under the high salt stress condition remains unknown. Here, we determined its complete genome sequence with the embedded regulatory elements and analyzed the transcriptional changes in response to a high-salt environment. Complete genome sequencing revealed a 3.70 mega base pair genome and three plasmids with a total of 3,589 genes annotated. Differential RNA-seq and Term-seq data aligned to the complete genome provided genome-wide information on genetic regulatory elements, including promoters, ribosome-binding sites, 5′- and 3′-untranslated regions, and terminators. Comparison with freshwater *Synechocystis* species revealed *Synechocystis* sp. PCC 7338 genome encodes additional genes, whose functions are related to ion channels to facilitate the adaptation to high salt and high osmotic pressure. Furthermore, a ferric uptake regulator binding motif was found in regulatory regions of various genes including SigF and the genes involved in energy metabolism, suggesting the iron-regulatory network is connected to not only the iron acquisition, but also response to high salt stress and photosynthesis. In addition, the transcriptomics analysis demonstrated a cyclic electron transport through photosystem I was actively used by the strain to satisfy the demand for ATP under high-salt environment. Our comprehensive analyses provide pivotal information to elucidate the genomic functions and regulations in *Synechocystis* sp. PCC 7338.

## Introduction

Cyanobacteria are the bacteria that generate energy through oxygenic photosynthesis (Camsund and Lindblad, 2014; Hitchcock et al., 2020). In addition to the photosynthetic capability, cyanobacteria, owing to their rapid growth and applicability in genetic engineering, are considered as potential industrial hosts for the production of value-added biochemicals (Lindberg et al., 2010; Ducat et al., 2011; Lan and Liao, 2011; Oliver et al., 2013; Savakis et al., 2013; Singh et al., 2016). Among those cyanobacterial hosts, freshwater cyanobacteria *Synechocystis* sp. PCC 6803 has been intensively studied to understand its genetic traits and metabolic networks, thus providing insights for effective engineering of the strain (Mitschke et al., 2011; Kopf et al., 2014; Hernandez-Prieto et al., 2016; Jablonsky et al., 2016; Vavitsas et al., 2017). Compared to this, *Synechocystis* sp. PCC 7338 is a marine cyanobacterium that grows under stressful environmental conditions characterized by high salinity and high osmotic pressure. In a recent metabolic and lipidomic profiling study, *Synechocystis* sp. PCC 7338 was reported to contain more unsaturated fatty acids, phosphatidylglycerol, and amino acids than *Synechocystis* sp. PCC 6803, which was suggested to be the result of adapting to the high-salt environment (Noh et al., 2020). Considering that seawater accounts for approximately 96.5% of the water on earth, and is a promising alternative for large-scale cultivation of cyanobacteria (Cui et al., 2020), these features make *Synechocystis* sp. PCC 7338 an attractive host for the mass production of commercially valuable bioproducts. Although several marine *Synechococcus* species, such as *Synechococcus* sp. PCC 7002 and *Synechococcus* sp. PCC 11901, have been used as bioproduction hosts, no marine *Synechocystis* species has been used. *Synechococcus* is an obligate photoautotroph, whereas *Synechocystis* is a facultative photoautotroph and has the advantage of increasing biomass and bioproduction in the presence of glucose (Varman et al., 2013). Moreover, it has been shown that Synechocystis is exceptional at growth and nutrient removal in waste treatment owing to the mixotrophic nature (Trentin et al., 2019). However, essential information on utilizing *Synechocystis* sp. PCC 7338 as an engineering host, including its genome sequence and regulatory elements is yet to be unraveled.

With the development of advanced next-generation sequencing techniques, recent studies have provided genome-wide information on the bacteria. In addition to the RNA-seq method that allows a systematic measurement of the transcriptome changes within the cells, techniques for cataloging the genome architecture have also been developed. In particular, the differential RNA-seq (dRNA-seq) method enabled the identification of the transcription start sites (TSSs), thereby revealing diverse regulatory elements, such as the promoters, 5′-untranslated regions (5′-UTRs), and small RNAs (sRNAs) (Qiu et al., 2010; Sharma et al., 2010; Kim et al., 2012; Jeong et al., 2016). Term-seq method has also been proven to be an effective tool for identifying genome-wide transcript 3′-end positions (TEPs) and 3′-untranslated regions (3′-UTRs) in various organisms (Dar et al., 2016a,b; Lee et al., 2019). In this study, the genome architecture and regulatory features of *Synechocystis* sp. PCC 7338 were identified with the detection of genome-wide TSSs and TEPs. We also describe the differences in the gene sets and the unique energy generation strategy in *Synechocystis* sp. PCC 7338 that facilitates its habitat adaptation compared to freshwater *Synechocystis* sp. PCC 6803.

## Materials and Methods

### Cell Growth

*Synechocystis* sp. PCC 7338 and *Synechocystis* sp. PCC 6803 cells were cultured under continuous illumination at 30 μmol photons m^–2^ s^–1^ at 30°C, and aerated with 2% CO_2_ balanced air (flow rate of 0.1 V–V^–1^ min^–1^). *Synechocystis* sp. PCC 7338 cells were cultured in ASN-III medium composed of 3.50 g MgSO_4_⋅7H_2_O, 2.00 g MgCl_2_⋅6H_2_O, 0.50 g CaCl_2_⋅2H_2_O, 0.75 g NaNO_3_, 0.015 g K_2_HPO_4_, 0.04 g Na_2_CO_3_, 0.003 g citric acid, 0.003 g (NH_4_)_5_Fe citrate, 0.50 g KCl, 25.00 g NaCl, 0.0005 g EDTA K_2_Mg⋅2H_2_O, and 1 mL of trace metal in 1 L distilled water. The composition of trace metal was as follow: 2.86 g H_3_BO_3_, 1.81 g MnCl_2_⋅4H_2_O, 0.22 g ZnSO_4_⋅7H_2_O, 0.39 g Na_2_MoO_4_⋅2H_2_O, 0.079 g CuSO_4_⋅5H_2_O, and 0.049 g Co(NO_3_)_2_⋅6H_2_O in 1 L distilled water. The pH of the ASN-III medium was adjusted to 7.4. Cells in the mid-exponential phase of growth (fresh cell weight of 0.91 ± 0.051 gl^–1^) were harvested for library construction. *Synechocystis* sp. PCC 6803 cells were cultured in BG-11 medium, and cells in the mid-exponential phase of growth (optical density at 730 nm = 0.8) were harvested for library construction.

### Genomic DNA and RNA Extraction

For genomic DNA extraction, the cells were collected by centrifugation at 4°C for 10 min at 3,000 × g, and the cell pellet was resuspended in 1 mL of lysis buffer composed of 10 mM Tris-HCl (pH 7.6), 5 mM MgCl_2_, and 40 mM NaCl. The resuspended cells were then dripped into a mortar filled with liquid nitrogen and grounded with a pestle. The powdered cells were thawed, and the cell debris was removed by centrifugation at 4°C for 5 min at 3,000 × g. The supernatant was further clarified and collected by centrifugation at 4°C for 10 min at 16,000 × g. The collected lysate was used for construction of the genome sequencing library. Genomic DNA was prepared using a genomic DNA extraction kit (Promega) according to the manufacturer’s protocol. For RNA-seq, dRNA-seq, and Term-seq library construction, the cells were resuspended in 1 mL solution composed of 25 mM Tris-HCl (pH 8.0), 10 mM EDTA, 50 mM glucose, and 2 mg/mL lysozyme (Sigma-Aldrich) and incubated at 30°C for 10 min. The cell pellet was collected by centrifugation at 4°C for 10 min at 16,000 × g, and then resuspended in 1 mL ice-cold solution composed of 50 mM sodium acetate (pH 5.3) and 10 mM EDTA. The resuspended sample was mixed with 100 μL of 10% sodium dodecyl sulfate (SDS) and 1.2 mL of phenol-chloroform mixture in the ratio of 5:1. The sample was incubated at 65°C for 5 min with periodic vortexing every 1 min. After centrifugation at 4°C for 20 min at 16,000 × g, 700 μL supernatant was mixed with 700 μL phenol-chloroform (5:1) solution. The sample was centrifuged at 4°C for 20 min at 16,000 × g, and the supernatant was used for total RNA extraction by isopropanol precipitation. To remove genomic DNA, the isolated RNA was incubated at 37°C for 1 h with 2 U of DNase I (New England Biolabs) and 5 μL of 10 × DNase I buffer (New England Biolabs). The RNA devoid of any DNA was purified by phenol-chloroform extraction and ethanol precipitation. Ribosomal RNA (rRNA) was removed using the Ribo-Zero rRNA Removal Kit (Epicenter) according to the manufacturer’s protocol.

### Genome Sequencing Library Preparation and Next-Generation Sequencing

The genome sequencing library for long-read sequencing was constructed by following the PacBio 20-kb library preparation protocol (Pacific Biosciences), and the library for short-read sequencing was constructed using the TruSeq DNA Sample Prep Kit (Illumina), according to the manufacturer’s protocol. Sequencing was performed using PacBio RS II with P6-C4 chemistry for the long-read sequencing library, and with the Illumina MiSeq v2 instrument with 1 × 50 bp read length for the short-read sequencing library. *De novo* assembly was conducted using the hierarchical genome assembly process workflow (HGAP v2.3), generating four contigs including one complete genome and three plasmids. The draft assemblies were improved by error correction using the Pilon software (Walker et al., 2014). The genes were annotated with the latest version of the National Center for Biotechnology Information (NCBI) Prokaryotic Genome Annotation Pipeline. For phylogenetic analysis, genome sequences of cyanobacteria were downloaded from the NCBI genome portal. The phylogenetic tree was generated by calculating the distance with the Up-to-date Bacterial Core Gene (UBCG) analysis pipeline and represented using the Randomized Axelerated Maximum Likelihood (RAxML) program. For the pan-genome analysis with other *Synechocystis* sp. strains, PGAP v1.12 program was used with the Gene Family (GF) method (Zhao et al., 2012). To find orthologs in *Synechocystis* sp. PCC 6803, total CDSs were compared using BLASTP search (*E*-value < 1.00 × 10^–6^). BLAST hits covering over 80% length of the CDS remained and were considered to be orthologs.

### RNA-Seq Library Preparation and Next-Generation Sequencing

RNA-seq libraries were constructed using TruSeq Stranded mRNA LT Sample Prep Kit (Illumina) according to the manufacturer’s protocol. The amplified library was purified using AgencourtAMPure XP beads (Beckman Coulter) and quantified using a Qubit 2.0 fluorometer (Invitrogen). The quantified libraries were sequenced using Illumina HiSeq 2000 platform with 1 × 50 bp read length.

### Differential RNA-Seq (dRNA-Seq) Library Preparation and Next-Generation Sequencing

The rRNA-depleted RNAs were split into two samples to construct two different libraries: a library of whole transcriptome (TAP +) and a library without primary transcripts (TAP −). To construct the TAP + library, 20 U of RNA 5′-polyphosphatase (Epicenter) was treated with 2 μL of 10 × RNA 5′-polyphosphatase Reaction buffer (Epicenter) and 20 U of SUPERase. In (Invitrogen) at 37°C for 1 h. RNA 5′-polyphosphatase converts the triphosphates at the 5′-end of the primary transcript to monophosphate and sequentially enables the ligation of 5′-RNA adaptor. For the TAP − library, RNA 5′-polyphosphatase was excluded from the reaction. After the RNA was purified by ethanol precipitation 0.5 μL of 10 μM 5′-RNA adaptor (5′- ACACUCUUUCCCUACACGACGCUCUUCCGAUCU -3′) was added to the purified mRNA with 10 U of T4 RNA Ligase (Thermo), 2 μL 10 × ligation buffer for T4 RNA Ligase (Thermo), and 0.1 mgmL^–1^ bovine serum albumin (BSA). The ligation reaction was incubated at 37°C for 90 min, and then the adaptor-ligated RNA was purified using AgencourtAMPure XP beads. The purified RNA was incubated with random 3′ overhanging primer (5′- GTGACTGGAGTTCAGAC GTGTGCTCTTCCGATCTNNNNNNNNN -3′) and 1 μL of 10 mM dNTPs (Invitrogen) at 65°C for 10 min. After the sample was chilled on ice, 2 μL of 10 × reverse transcription (RT) buffer, 2 μL of 100 mM dithiothreitol (DTT), 4 μL of 25 mM MgCl_2_, 20 U of SUPERase.In, and 200 U of SuperScript III Reverse Transcriptase (Invitrogen) were added followed by incubation at 25°C for 10 min, 50°C for 1 h, 85°C for 5 min, and 4°C for chilling, sequentially. To remove residual RNAs, 2 U of RNase H (Invitrogen) was added to the reaction, and the mixture was incubated at 37°C for 20 min. The synthesized complementary DNA (cDNA) was purified using AgencourtAMPure XP beads, and amplified by polymerase chain reaction (PCR) with the indexed primer for Illumina sequencing. The amplification step was monitored on a CFX96 Real-Time PCR Detection System (Bio-Rad) before the PCR reaction was fully saturated. Finally, the amplified library was purified using AgencourtAMPure XP beads, and the concentration of the library was measured with Qubit 2.0 fluorometer. The size distribution of the library was checked by 2% agarose gel electrophoresis. The constructed dRNA-seq library was sequenced by using Illumina HiSeq 2000 platform with 1 × 50 bp read length.

### Term-Seq Library Preparation and Next-Generation Sequencing

Term-seq library was prepared as described in a previous study with some modifications (Dar et al., 2016b). Briefly, modified DNA adaptor (5′- NNAGATCGGAAGAGCGTCGTGT -3′) was ligated to the 3′ end of the mRNA. The adaptor-ligated mRNA was purified using AgencourtAMPure XP beads followed by fragmentation with 10 × fragmentation buffer (Ambion). The fragments were purified with AgencourtAMPure XP beads, and then reverse transcribed using SuperScript III Reverse Transcriptase. The synthesized cDNA was then purified with AgencourtAMPure XP beads, cDNA 3′-adaptor (5′- NNAGATCGGAAGAGCACACGTCTGAACTCCAGTCAC -3′) was ligated to the 3′ end of the cDNA. The adaptor-ligated cDNA was purified using AgencourtAMPure XP beads. For amplification, the indexed primer for Illumina sequencing was used, and the amplification step was monitored on a CFX96 Real-Time PCR Detection System. The amplified library was removed from the PCR machine at the beginning of the saturation point, and purified using AgencourtAMPure XP beads. The concentration and size distribution of the final library were checked with a Qubit 2.0 fluorometer and an Agilent 2200 TapeStation System (Agilent), respectively. The Term-seq library was sequenced by using Illumina HiSeq 2000 platform with 1 × 50 bp read length.

### Quantitative Real-Time PCR

cDNAs were synthesized from rRNA-depleted RNAs by using SuperScript III First-Strand Synthesis System (Invitrogen) according to the manufacturer’s instructions. KAPA SYBR^®^ FAST qPCR Master Mix (KAPA Biosystems) and StepOnePlus Real-Time PCR System (ThermoFisher) was used to measure the different expression level of these genes. The expression fold changes were calculated from the Ct values. The primers used for amplification are indicated in Supplementary Table 1.

### Data Processing

The adaptor sequences were trimmed from the reads of the dRNA-seq and Term-seq libraries. Random (N) sequences from dRNA-seq and Term-seq reads were also trimmed. After trimming, the reads shorter than 15 bp were discarded and the remaining reads were aligned to the genome of *Synechocystis* sp. PCC 7338 using CLC genomics workbench with the following parameters: mismatch cost = 2, deletion cost = 3, insertion cost = 3, length fraction = 0.9, and similarity fraction = 0.9. Only uniquely mapped reads were retained. The 8.8–11.4 million sequence reads were mapped to the reference genomes of *Synechocystis* sp. PCC 6803 and PCC 7338 with at least 99 × and 109 × coverage, respectively. To visualize the data with SignalMap (Roche Nimblegen), the read depth of each genomic position of each library was normalized by multiplying a size factor (one million divided by the total number of reads in each library). To compare the mRNA expression of orthologs between the two strains, a size factor was applied to the number of reads mapped to the orthologs, and thereafter the gene expression was normalized with DESeq2 package in R (Love et al., 2014). The reproducibility between biological triplicates was confirmed by drawing a heatmap and a principal component analysis (PCA) plot (Supplementary Figures 4B,C). Compared to the mRNA expression of orthologs in *Synechocystis* sp. PCC 6803, genes with over 2-fold changes in mRNA levels and adjusted *p* < 0.01, were selected as DEGs.

### TSS Identification and Data Analysis

The 5′ end positions of the reads of TAP-treated (TAP +) libraries were considered as potential TSSs. The depths of the reads in each library were normalized by multiplying it with a size factor. Next, the potential TSSs were clustered if the distance between them were <100 nt. To subdivide the clusters, the standard deviation in the peak positions within a cluster was calculated. A standard deviation of <10 for two or more nearby located peaks indicated a region with densely located peaks. Thus, the peaks were sub-clustered together and the one with highest depth in the sub-cluster remained. The remaining peaks were compared with data from the respective non-TAP-treated (TAP −) libraries. The peaks that only existed in the TAP + library or had over 2-fold depth than the peak of the TAP − library remained. If the remaining peak was absent in one of the biological duplicate data, the peak was removed. Further manual curation was performed to finalize the TSS list by comparing the peaks and dRNA-seq profiles with RNA-seq profiles. If a peak was removed due 1–2 nt difference between the biological duplicate data, the peak was restored and selected as a TSS. In addition, if a peak was present in one of the biological duplicate data and had a clear RNA-seq profile, it was selected as a TSS. In contrast, if the expression level of a gene was extremely high, it was difficult to remove the peaks of processed transcripts by the above curation steps; thus, we removed the peaks by manual curation. Among the TSSs located within 500 nt upstream to 100 nt downstream of the start codon of a gene, the one with the highest read depth was categorized as primary (P) TSS, while the others were categorized as secondary (S) TSSs. The TSSs located inside or the antisense orientation of a gene were categorized as internal (I) TSSs or antisense (A) TSSs, respectively. The TSSs that did not belong to any of these categories were considered intergenic (N) TSSs. To calculate the 5′-UTR sequence difference from the 5′-UTR of *Synechocystis* sp. PCC 6803, the 5′-UTR sequences were aligned by MUSCLE, and the distance between each sequence was calculated using the p-distance method with pairwise deletion option in MEGA X software (Kumar et al., 2018). The 5′-UTR lengths were compared for 397 genes with 5′-UTR identified in both the strains; Those having length differences under 2 nt were considered as conserved 5′-UTRs, and length differences over 10 nt as degenerated 5′-UTRs. For comparison of the promoter region, the 50 nt upstream sequences (promoter region) of 754 TSSs associated with the CDSs of *Synechocystis* sp. PCC 7338 were searched against the 550 nt upstream sequence of ortholog from *Synechocystis* sp. PCC 6803 by BLASTN. Conversely, the 50 nt upstream sequences of 1,384 TSSs associated with the CDSs of *Synechocystis* sp. PCC 6803 were searched against the 550 nt upstream sequence of ortholog from *Synechocystis* sp. PCC 7338 by BLASTN. If there was a matched promoter region with associated TSS from both search results, the promoter region was classified as ‘conserved’. If there was a matched promoter region in the upstream sequence of orthologs from each other with no TSS or mismatched TSS position, the promoter region was classified as ‘mismatched’. If there was no matched promoter region in the upstream sequence of ortholog from each other, the promoter region was classified as ‘orphan’. Finally, if the promoter region is associated with the genes that have no ortholog, it was classified as ‘specific’.

### TEP Identification and Data Analysis

The 5′end positions of the Term-seq library reads were extracted and the strand information of the 5′end positions were reversed to be considered as potential TEPs. The TEPs were determined by a combined method of previous studies with a machine-learning algorithm (Dar et al., 2016b; Lalanne et al., 2018). First, to select the positive control learning set, the clustering method used for TSS identification was used. The selected peaks were curated by calculating the Z-score of their read depth (*Z*-score >6), which indicated the enrichment of the peak compared to adjacent peaks. Among the remaining peaks, the positive control learning set was manually selected by considering the decreasing RNA expression profiles near the peaks. The negative control learning set was selected within the peaks located at ± 10 nt position of the positive control learning sets. Using the positive and negative control sets, the TEPs were identified with an in-house Python script based on the scikit-learn package. Briefly, for a reversed 5′ end position, which is a potential TEP, the read depth in ± 10 nt positions were submitted to the K-nearest neighbor (KNN) machine classifier. The Python script is available at http://cholab.or.kr. Finally, only the TEPs present in at least two or more of the triplicate data (± 1 nt) remained and were manually curated by considering the RNA expression profiles near them. Among the TEPs located within 500 nt downstream of the stop codon, the one with the highest depth was classified as primary P-TEP, and the others were classified as secondary S-TEPs. Based on the criteria, 329 P-TEPs and 25 S-TEPs were assigned. If a TEP was located between the start codon and the TSS assigned to the gene, it was classified as 5′-UTR TEP. There were 28 genes with 5′-UTR TEPs at their 5′-UTRs. A TEP located inside a gene was classified as internal I-TEP, and one located in the antisense strand of a gene was classified as antisense A-TEP. Finally, the remaining TEPs were classified as intergenic N-TEPs. The TEPs were classified as L-shaped TEPs if there were ≥4 uridines near TEP, and I-shaped TEPs if there were <4 uridines. For comparison of the terminator regions, we downloaded and analyzed the Term-seq data of *Synechocystis* sp. PCC 6803 deposited in NCBI Sequence Read Archive (Cho and Jeong, 2020). The same strategy for the comparison of the promoter region was applied with modifications to compare and classify the terminator regions; the terminator regions were considered as 40 nt upstream to 20 nt downstream sequences of the TEPs, and they were BLASTN searched against the 520 nt downstream of the orthologs.

### Motif Discovery and ΔG_*folding*_ Calculation

The conserved sequences were examined using the MEME suite (Bailey et al., 2009). The 20 nt upstream sequences of each TSS and the sequences between 40 nt and 20 nt upstream of each TSS were used for searching the − 10 promoter motif and the − 35 promoter motif, respectively. RBS was searched in 20 nt to 1 nt upstream sequences of start codons of genes that have 5′-UTR lengths >10 nt. The 40 nt upstream to 20 nt downstream sequences of each TEP were used for searching the terminator sequences. We extracted the conserved sequences and obtained the motif sequences with WebLogo (Crooks et al., 2004). The ΔG_*folding*_ values from the upstream sequences of TEPs were predicted using RNAfold (Lorenz et al., 2011).

### Accession Numbers

The datasets, genome-seq, RNA-seq, dRNA-seq, and Term-seq, generated for this study have been deposited in National Center for Biotechnology Information (NCBI) as BioProject PRJNA629670^1^.

## Results

### Genome Completion and Annotation

**FIGURE 1:**
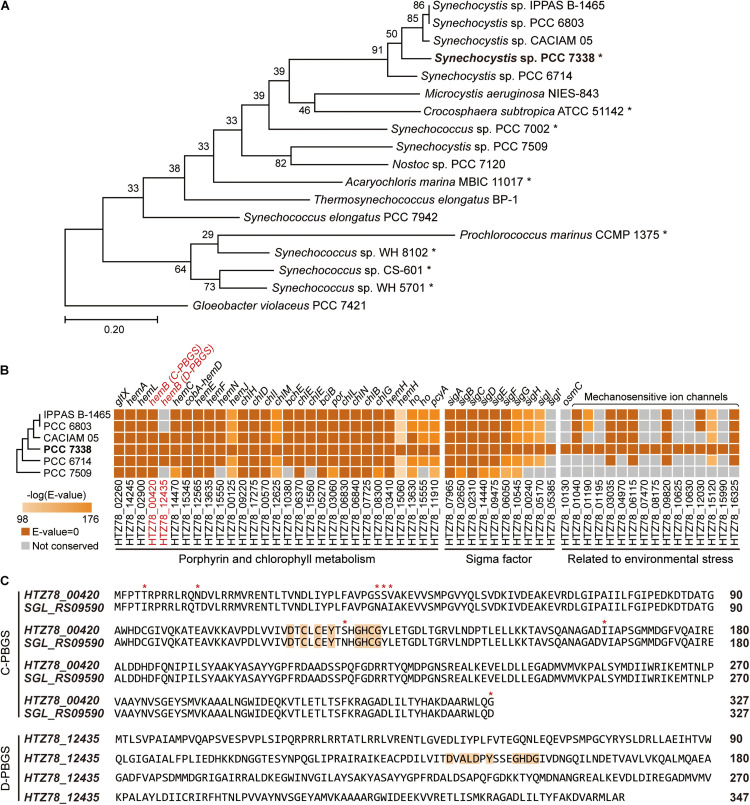
Genome completion of *Synechocystis* sp. PCC 7338 and comparison with other cyanobacteria. **(A)** Phylogenetic analysis of *Synechocystis* sp. PCC 7338 and 12 genome sequenced cyanobacteria. *Gloeobacter violaceus* PCC 7421 was selected as the outgroup. The evolutionary distances were calculated by Up-to-date Bacterial Core Gene analysis pipeline (UBCG) and represented by Randomized Axelerated Maximum Likelihood (RAxML). Asterisks indicate marine cyanobacteria. **(B)** Comparison of gene sequences related to porphyrin and chlorophyll metabolism, sigma factors, and environmental stress in six *Synechocystis* sp. strains by BLASTP search. The names of strains are indicated with omitted species name (*Synechocystis* sp.). The extra sigma factor detected only in *Synechocystis* sp. PCC 7338 was denoted as SigI’. The tree is not to scale. Keys: C-PBGS, cysteine-rich porphobilinogen synthase; D-PBGS, aspartate-rich porphobilinogen synthase. **(C)** The amino acid sequences of C-PBGS in *Synechocystis* sp. PCC 7338 (HTZ78_00420) and *Synechocystis* sp. PCC 6803 (SGL_RS09590) and D-PBGS in *Synechocystis* sp. PCC 7338 (HTZ78_12435). The amino acid sequences of the other *Synechocystis* species are compared in Supplementary Figure 1B. Orange boxes indicate the active sites and red asterisks indicate the varying sequences.

To obtain a high-quality genome sequence of *Synechocystis* sp. PCC 7338, we utilized two sequencing platforms, the PacBio and Illumina providing long read and short read outputs, respectively. The sequencing reads were assembled to obtain the complete genome sequence without any sequence gaps, resulting a 3.70 mega base pair (Mbp) circular chromosome and three plasmids of sizes 81.33 kilo base pair (kbp), 45.22 kbp, and 1.56 kbp (Supplementary Table 2). The benchmarking universal single-copy orthologs (BUSCO) analysis showed 98.39% of complete BUSCO, 1.61% of fragmented BUSCO, and no missing BUSCO in *Synechocystis* sp. PCC 7338 genome, validating that the completed genome sequence was of high-quality (Simao et al., 2015). Compared to the genome sequence of model cyanobacteria, *Synechocystis* sp. PCC 6803, the calculated average nucleotide identity (ANI) value was 86.96, which indicates that they are closely related species with moderately divergent genomes (Jain et al., 2018). The phylogenetic analysis revealed a close relationship between *Synechocystis* sp. PCC 7338 and other freshwater *Synechocystis* sp. strains, in addition to similar sizes and GC contents of the genomes (Figure 1A and Supplementary Table 2).

Next, of the 3,589 genes annotated in the complete genome sequence, 3,385 were coding sequences (CDSs) with 152 pseudogenes, 42 transfer RNAs (tRNAs), six ribosomal RNAs (rRNAs), and four RNAs (Supplementary Table 2). The annotated CDSs were categorized by the functions based on Kyoto Encyclopedia of Genes and Genomes (KEGG) Orthology (KO). Among the 3,385 CDSs, 2,573 KO IDs were annotated, and a large number of genes were involved in carbohydrate metabolism (17.27%), energy metabolism (14.01%), amino acid metabolism (12.84%), and metabolism of cofactors and vitamins (11.97%) (Supplementary Figure 1A). Particularly, in *Synechocystis* sp. PCC 7338, the energy metabolism genes were highly enriched owing to the presence of photosynthesis-associated genes, similar to other photosynthetic organisms (Nakayama et al., 2014).

### Additional Genes in *Synechocystis sp.* PCC 7338 Compared to Freshwater *Synechocystis sp.* Strains

We compared genes in *Synechocystis* sp. PCC 7338 with those in other freshwater *Synechocystis* sp. strains to investigate the differences in gene sets that enables them to survive in different environmental conditions. The genome information of six *Synechocystis* sp. strains were subject to pan-genome analysis, revealing 396 specific genes in *Synechocystis* sp. PCC 7338, which can be responsible for the distinctive characteristics of the strain (Supplementary Table 3). Interestingly, there were core or dispensable genes that were strain-specifically duplicated, which can also describe evolutionary characteristics. For example, while three species possessed two copies of *hemB*, which encode porphobilinogen synthase (PBGS) involved in the porphyrin and chlorophyll metabolism pathways, the rest had a single copy of *hemB* (Figure 1B). The PBGS exhibits phylogenetic variation according to the active site sequence, which is suggested to evolve from being cysteine-rich (C-) to aspartate-rich (D-) PBGS (Jaffe, 2003). Interestingly, between the two *hemB* genes in *Synechocystis* sp. PCC 7338 genome, one encoded C-PBGS and the other D-PBGS (Figure 1C and Supplementary Figure 1B). To date, only *Nostoc* sp. PCC 7120 has been reported to express both types of PBGS (Jaffe, 2003). It is noteworthy that although cyanobacteria are the ancestors of plant plastids, the plants express only D-PBGS (Jaffe, 2003). Thus, cyanobacteria expressing both C- and D-PBGSs may be involved in the evolution of C-PBGS to D-PBGS and thus closely resembling the plant plastids. When the transcription levels of the two genes were compared by using RNA-seq data (see “Materials and Methods”), gene expression of C-PBGS was 13.5-fold higher than that of D-PBGS (Supplementary Figure 1C). Despite the existence of both type of PBGSs, the different transcription levels infer that *Synechocystis* sp. PCC 7338 mainly uses C-PBGS, and presumably has D-PBGS as a trace of evolution.

*Synechocystis* sp. PCC 7338 is presumed to possess specific genes that confer the ability to withstand high salinity and high osmotic pressure. Those are osmotically inducible protein C (OsmC) family of proteins (*HTZ78_10130*) and six additional mechanosensitive ion channels (*HTZ78_01195*, *HTZ78_07470*, *HTZ78_08175*, *HTZ78_10625*, *HTZ78_10630*, and *HTZ78_15990*) (Figure 1B and Supplementary Table 4). The OsmC accumulates under osmotic stress, and the mechanosensitive ion channels regulate the turgor in bacteria in response to changes in osmotic pressure (Perozo and Rees, 2003). Thus, *Synechocystis* sp. PCC 7338 has more mechanosensitive ion channels than freshwater *Synechocystis* sp. strains to respond to the high osmotic pressure. Also, we observed an additional sigma factor and an anti-sigma factor present exclusively in *Synechocystis* sp. PCC 7338 (Figure 1B and Supplementary Table 4). Among the annotated sigma factors in *Synechocystis* sp. PCC 7338 genome, seven (SigA, SigB, SigC, SigD, SigE, SigF, and SigG) were conserved in five other *Synechocystis* sp. strains, and two (SigH and SigI) were conserved in four strains (Figure 1B). Interestingly, one sigma factor, encoded by *HTZ78_05385*, was not found in any *Synechocystis* sp. strains. Among the annotated sigma factors, SigI encoded by *HTZ78_05170* had the highest similarity to *HTZ78_05385* with a 50.24% identity (BLASTP *e* = 2.00 × 10^–52^) and similar amino acid lengths (185 and 187, respectively) (Supplementary Figure 1D). The evolution of cyanobacterial SigI was believed to be strain-specific, which responded rapidly under specific stress conditions, contributing to cell survival (Imamura and Asayama, 2009). Thus, it suggests that *HTZ78_05385* encodes a strain-specific sigma factor in *Synechocystis* sp. PCC 7338, which responds to specific environmental stress conditions.

### Identification of TSSs, Promoters, and 5′-UTRs

**FIGURE 2:**
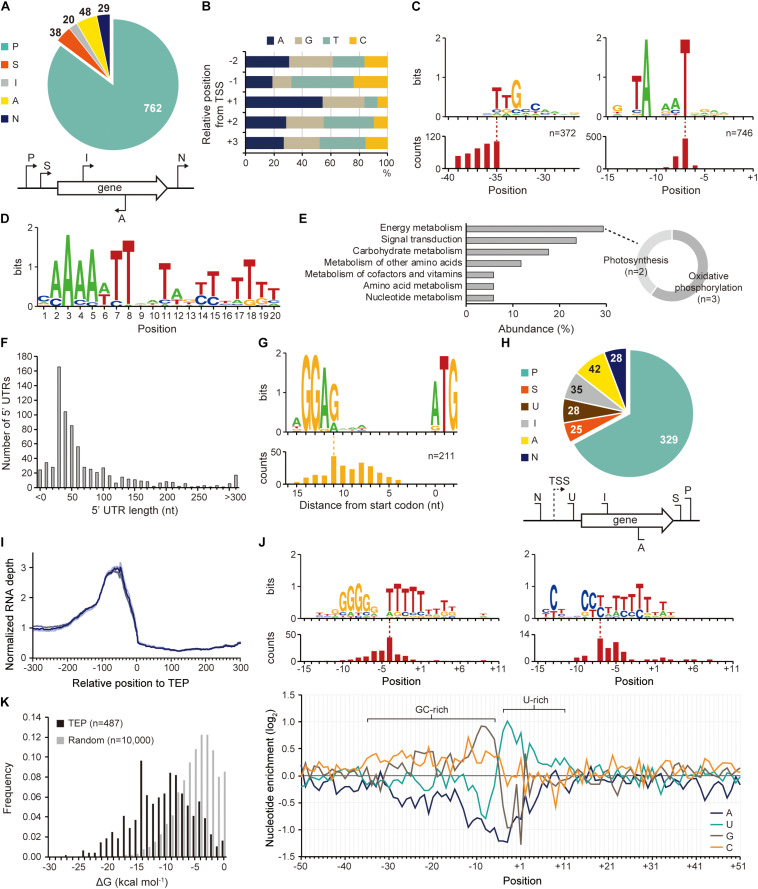
Identification of transcription start site (TSS), transcript 3′-end position (TEP), and regulatory elements involved in transcription regulation. **(A)** TSS categorization by their relative positions to adjacent genes. **(B)** The nucleotide frequency calculated near the TSSs shows purine preference at TSS (+ 1 position) and pyrimidine preference at – 1 position. **(C)** Conserved – 10 and – 35 promoter motifs. The relative position of the motif to the TSS is represented at the bottom. **(D)** The Fur binding motif found at the upstream regions of *Synechocystis* sp. PCC 7338-specific genes. **(E)** The KEGG pathway analysis of the genes having the Fur motif. The sub-categories of the category with the highest abundance (energy metabolism category) were indicated as a doughnut chart. **(F)** The length distribution of 5′-untranslated region (5′-UTR). **(G)** The ribosome binding site (RBS) sequence detected at 5′-UTR. The relative position of the RBS to the start codon is represented at the bottom. **(H)** TEP categorization by their relative positions to adjacent genes. Keys: P, primary TEP; S, secondary TEP; U, 5′-UTR TEP; I, internal TEP; A, antisense TEP; N, intergenic TEP. **(I)** RNA expression profiles near the identified TEPs. Each line shows each result from triplicate RNA-seq data. **(J)** The top panel shows conserved sequences detected near the TEPs. The relative positions of the sequences to the TEPs are represented at the bottom of each sequence. The bottom panel shows nucleotide enrichment calculated in ± 50 nt sequences from TEPs. The ratio of each nucleotide at each position was normalized with those of randomly selected intergenic positions (*n* = 10,000). **(K)** The folding energy was calculated at upstream sequences of TEPs or at randomly selected intergenic positions.

The existence of an exclusive strain-specific sigma factor suggests the presence of specific regulatory networks in *Synechocystis* sp. PCC 7338, which are involved in the stress conditions. To examine the regulatory traits, we identified the TSS positions in *Synechocystis* sp. PCC 7338 genome using dRNA-seq. A total of 897 TSSs were identified and categorized based on their positions relative to the annotated genes (see Materials and Methods for details) (Figure 2A and Supplementary Data 1). The dinucleotide preference, which is high purine usage at the TSS position (84.62%) and high pyrimidine usage at the − 1 position (67.56%), was observed, similar to other previously reported bacteria (Figure 2B; Jeong et al., 2016; Hwang et al., 2019; Lee et al., 2019). Given the significance of the promoter motifs in regulating the transcription initiation step, we next investigated the conserved promoter motifs from the upstream sequences of TSSs. Among the 897 TSSs, − 10 promoter and − 35 promoter motif sequences were detected as 5′-TANAAT and 5′-TTGCCAA in the upstream regions of 746 TSSs and 372 TSSs, respectively (Figure 2C). Similar motif sequences were detected in *Synechocystis* sp. PCC 6803 by searching the upstream sequence of previously published TSSs (Supplementary Figure 2A) (Mitschke et al., 2011; Kopf et al., 2014).

Interestingly, we found an AT-rich ferric uptake regulator (Fur) binding motif in the upstream sequences of 77 genes, including 43 specific genes in *Synechocystis* sp. PCC 7338, by MEME and FIMO search (Figure 2D and Supplementary Table 5) (Bailey et al., 2009; Grant et al., 2011). Fur is involved in iron homeostasis by regulating several iron responsive genes (Dang et al., 2012; Kaushik et al., 2016). The Fur binding motif was found at the TSS upstream regions of the genes encoding bacterioferritin (HTZ78_10445), which is a ferritin-type storage complex, and PerR (HTZ78_00325), which is a Fur family transcriptional regulator/peroxide stress response regulator (Supplementary Table 5; Shcolnick et al., 2009). Among the genes having the Fur motif sequence and KEGG orthology identifier, five were included in the energy metabolism category (29.41%), containing the photosynthesis and oxidative phosphorylation categories, consistent with the high iron requirement for photosynthesis in cyanobacteria (Figure 2E; Xie et al., 2011; Morrissey and Bowler, 2012). In addition, four genes were included in the signal transduction category, two of which were regulators involved in pilus function: *pilH* (HTZ78_05555) encoding a twitching motility two-component system response regulator and *chpA* (HTZ78_02660) encoding a chemosensory pili system protein (sensor histidine kinase/response regulator) (Figure 2E and Supplementary Table 5). Interestingly, SigF, a sigma factor known to regulate pili gene expression in cyanobacteria, also has the Fur binding motif (Imamura and Asayama, 2009). These results agree with the previous reports suggesting that pili are involved in the reduction of iron oxides in bacteria, including *Synechocystis* sp. PCC 6803 (Lamb et al., 2014). SigF is also known to be involved in the long-term application of high salt stress in *Synechocystis* sp. PCC 6803 (Huckauf et al., 2000). In addition, an extracytoplasmic function (ECF) sigma factor SigG was found to have the Fur binding motif in its TSS upstream sequence. SigG showed high similarities to two ECF sigma factors of *Escherichia coli*, RpoE and FecI, which are involved in strong heat shock response and iron uptake regulation, respectively (Huckauf et al., 2000). Thus, the Fur binding motif indicates the possibility that the sigma factor is regulated by iron concentration, and orchestrates the regulatory network involved in iron homeostasis in *Synechocystis* sp. PCC 7338.

The SigG and other ECF sigma factors (SigH, SigI, and HTZ78_05385) were further analyzed to investigate their functions in stress responses. The amino acid sequences of region 2 and region 4, which are the conserved domains of ECF sigma factors, were aligned with previously known bacterial ECF sigma factors (Todor et al., 2020). In results, it was revealed that both region 2 and region 4 sequences of SigG, SigH, and SigI of *Synechocystis* sp. PCC 7338 were close to those of *Synechocystis* sp. PCC 6803 and *Synechocystis* sp. PCC 6714 (Supplementary Figure 2B) (Todor et al., 2020). On the other hand, the region 2 sequence of HTZ78_05385 was close to an ECF sigma factor of another marine cyanobacteria, *Nodularia spumigena*, inferring the function of HTZ78_05385 is related to the adaptation to marine environment. Considering the promoter motifs predicted for clusters of ECF sigma factors, which had been clustered by region 2 and region 4 sequences in the previous study, we found 5′-GTC of − 10 motif and 5′-GGAAC of − 35 motif for SigG, and the extended − 10 and − 35 motifs for SigH; SigI and HTZ78_05385 were predicted to have 5′-CGTA of − 10 motif and 5′-CATCC of − 35 motif, which are distinctive from those of SigG and SigH (Supplementary Figure 2C) (Todor et al., 2020).

The identified TSS positions provide information on the 5′-UTR, an important genetic element involved in the regulation of gene expression at transcriptional and post-transcriptional levels. Among the 737 5′-UTRs, 55.77% had a length varying between 20–59 nucleotides (nt), and the median length was 42 nt (Figure 2F). The conserved ribosome binding site (RBS) was identified within the 5′-UTRs as 5′-AGGAG, and major portion of the RBS (74.41%) was at a distance of 5–10 nt from the start codon (Figure 2G). The RBS sequence was not detected at the upstream sequence of the leaderless transcripts with a 5′-UTR length <9 nt (Supplementary Figure 2D). The 5′-AGGAG located at a distance of 5–10 nt from the start codon has been identified as the third most frequently used RBS sequence in bacteria (Omotajo et al., 2015).

### Identification of TEPs and 3′-UTRs

The 3′-UTR is also a crucial genetic element that affects gene expression, mRNA stability, transcription termination rate, and interaction with the RNA-binding proteins (Dar et al., 2016a,b). Using Term-seq coupled with machine-learning approach, a total of 487 TEPs were identified and categorized based on their relative positions to the adjacent genes (see Materials and Methods for details) (Figure 2H and Supplementary Data 2). To confirm the identified TEPs, we used RNA-seq data to investigate the RNA expression profiles near TEPs (Figure 2I). We observed that the RNA expression levels decreased steeply from 50 nt upstream of TEPs, supporting the reliability of the determined TEPs.

Next, the sequences near TEPs were analyzed to search for conserved sequences involved in the regulation of transcription termination. One of the searched motifs had a G-rich region followed by an uracil (U)-rich region, and the other one had a C-rich region followed by a U-rich region (Figure 2J). These motifs resemble the shape of a bacterial rho-independent terminator, which has a GC-stem and U-tract (Mitra et al., 2009). We observed that a similar motif was also found in *Synechocystis* sp. PCC 6803 (Supplementary Figure 2E). Since no homologs of the rho factor were identified in cyanobacteria, most transcription termination were suggested to occur via a rho-independent mechanism (Ramey et al., 2015). The patterns were also observed during the calculation of nucleotide enrichment near TEP, which is similar to other bacteria such as *E. coli* and *Streptomyces lividans* (Figure 2J; Dar and Sorek, 2018; Lee et al., 2019). The formation of a secondary structure at the upstream sequences of TEPs was investigated by calculating the free energy of folding (ΔG_*folding*_). The ΔG_*folding*_ distribution was relatively lower than that of random intergenic regions (*n* = 10,000), indicating that secondary structure formation upstream of TEP is more stable (Figure 2K).

Based on the shape of the rho-independent terminators in bacteria, we classified the identified TEPs into L- or I-shaped TEPs (Unniraman et al., 2002). The nucleotide enrichment near the L-shaped TEPs was similar to that of the total TEPs, whereas the I-shaped TEPs showed an omitted U-rich region and stretched GC-rich region (Supplementary Figure 2F). However, despite the broader distribution of the GC-rich region in the I-shaped TEPs, the ΔG_*folding*_ values were lower in the L-shaped TEPs, indicating that the secondary structures of the L-shaped TEPs were more stable (Supplementary Figure 2G). The RNA expression profile decreased from 100 nt upstream regions of L-shaped TEPs, and a sharper decrease was observed from 50 nt upstream regions of the I-shaped TEPs, suggesting both types of TEPs are the 3′-termini of the transcripts (Supplementary Figure 2H). Previously, the read-through effect of RNA polymerase at I-shaped TEP was reported in *S. lividans*, however, the read-through effect at the I-shaped TEP appears uncommon in *Synechocystis* sp. PCC 7338 (Lee et al., 2019).

### Regulatory Regions in *Synechocystis* sp. PCC 7338 Compared to *Synechocystis* sp. PCC 6803

**FIGURE 3:**
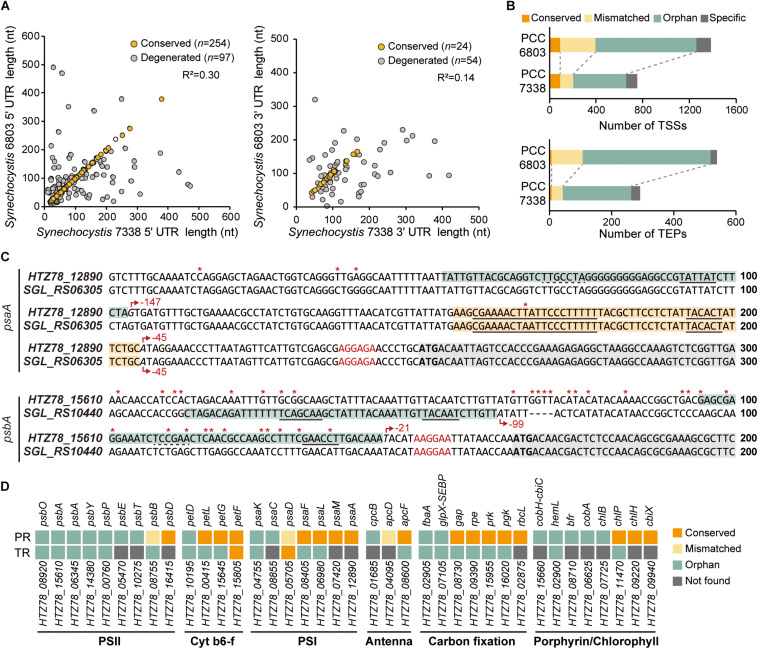
Comparison of the regulatory regions between *Synechocystis* sp. PCC 7338 and *Synechocystis* sp. PCC 6803. **(A)** The 5′-UTR and the 3′-UTR lengths of orthologs were compared with *Synechocystis* sp. PCC 6803. The UTR lengths were classified as conserved or degenerated according to the length difference between the two strains. **(B)** The promoter regions and the terminator regions were compared between the two species and classified as four categories. **(C)** Examples of the compared promoter regions. *psaA* has a conserved promoter region (orange boxes) and an orphan promoter region (cyan box), and *psbA* has orphan promoter regions (cyan boxes). Grey boxes indicate the coding sequences. Red asterisks indicate the varying sequences. Red arrows indicate the TSSs, and the relative positions of the TSSs to the start codons are designated near the red arrows. The promoter sequences detected from MEME search are underlined with solid lines, and the predicted promoter sequences are underlined with dotted lines. The RBS sequences are indicated as red characters, and the start codons are indicated as bold characters. **(D)** The classification of the regulatory regions associated with the genes related to photosynthesis. Keys: PR, promoter region; TR, terminator region, PSII, photosystem II; Cyt b6-f, cytochrome b6-f; PSI, photosystem I.

We compared the regulatory regions of the two strains to investigate the differences in gene regulation (Mitschke et al., 2011; Kopf et al., 2014; Cho and Jeong, 2020). From a total of 397 5′-UTR and 93 3′-UTR from orthologs in the two strains, 63.98% of the 5′-UTR pairs have conserved length, while 25.81% of the 3′-UTR pairs have conserved length (Figure 3A). We observed that > 27% of the orthologs with conserved 5′-UTR length was associated with carbohydrate metabolism, while > 30% of the orthologs with degenerated 5′-UTR length was involved in energy metabolism, especially photosynthesis (Supplementary Figure 3A).

Next, the promoter regions (50 nt upstream sequences of TSSs) of the orthologs in the two strains were compared (see Materials and Methods for details). The results were classified into four categories; the ones with conserved promoter regions and associated TSSs were classified as ‘conserved’, the ones with conserved promoter regions but mismatched TSSs as ‘mismatched’, the ones whose promoter regions were not conserved as ‘orphan’, and the ones associated with no orthologs as ‘specific’ (Figure 3B and Supplementary Data 3). For example, among the two TSSs associated with *HTZ78_12890* encoding photosystem I (PSI) core protein (PsaA), one of them has the promoter region conserved with its ortholog, *SGL_RS06305* (Figure 3C). On the other hand, the promoter region from the *HTZ78_15610* encoding photosystem II (PSII) protein D1 (PsbA) was not conserved with its ortholog, *SGL_RS10440*, and the TSS position also differed, thus making it an orphan promoter region (Figure 3C and Supplementary Figure 3B). Considering the previous result that 17.70% of the promoter regions were conserved between *E. coli* and *Klebsiella pneumoniae*, the lower ratio of the conserved promoter regions between the two *Synechocystis* species (12.47%) is noticeable (Kim et al., 2012). It can be inferred that the regulatory mechanism of the two species differentiated in a large degree to adapt to the different environmental conditions. In addition, among the genes related to photosynthesis, the genes involved in cytochrome b6-f complex, PSI, and carbon fixation tend to have more conserved promoter regions than PSII genes, suggesting that the regulatory regions of the PSII genes had diverged more between the two environments (Figure 3D).

For comparison of the terminator regions, the proximate sequences of the TEPs of the orthologs in the two strains were compared (see Materials and Methods for details). The overall characteristics such as ΔG_*folding*_ distribution and the nucleotide enrichment were similar between the two strains, however, only 2.40% of the compared terminator region were found to be conserved (Figure 3B and Supplementary Figure 3C). When considering that the 3′-UTR length is also less conserved than the 5′-UTR lengths, the terminator region seems to operate by the formation of the stem-loop structure rather than the precise sequence-mediated mechanism.

### Comparison of Gene Expression Patterns of *Synechocystis sp.* PCC 7338 With *Synechocystis sp.* PCC 6803

**FIGURE 4:**
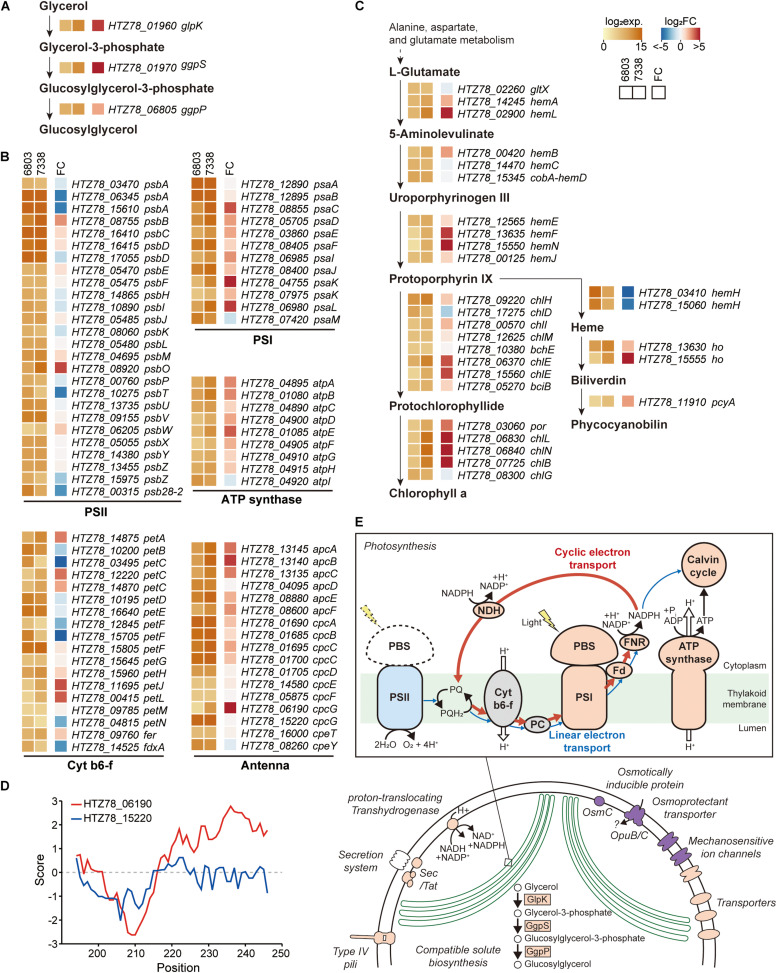
Messenger RNA (mRNA) expressions in *Synechocystis* sp. PCC 7338. **(A–C)** Log_2_ mRNA expression levels and log_2_ fold changes of orthologs in *Synechocystis* sp. PCC 7338 compared to those of *Synechocystis* sp. PCC 6803. The orthologs related to glucosylglycerol biosynthesis (**A**), photosynthesis including photosystem II (PSII), cytochrome b6-f (cyt b6-f), photosystem I (PSI), ATP synthase, and antenna proteins **(B)**, and porphyrin and chlorophyll metabolism pathway **(C)** were described. **(D)** Hydrophobicity calculated at the C-terminal of 2 *cpcG* in *Synechocystis* sp. PCC 7338. **(E)** Schematic illustration of proposed response in *Synechocystis* sp. PCC 7338. Orange color indicates the upregulation of the component compared to *Synechocystis* sp. PCC 6803, and blue color indicates the downregulation. Purple color indicates the specific component that is exclusively present in *Synechocystis* sp. PCC 7338.

Regulatory elements, such as promoters, 5′-UTRs, RBSs, 3′-UTRs, and terminators, are involved in gene expression regulation of bacteria confronting diverse environmental conditions. Although the two strains have 2,790 orthologs, their transcription levels vary given the differential growing environmental conditions. Thus, for comparison, we additionally performed RNA-seq in *Synechocystis* sp. PCC 6803 (Supplementary Figures 4A-C). The reads aligned to 2,790 orthologs in each strain were normalized and compared among each other, obtaining a list of 1,504 differentially expressed genes (DEGs) (Supplementary Data 4). We observed a significant upregulation of the key enzymes involved in the glucosylglycerol biosynthesis pathway, such as 19.6-fold increase in glycerol kinase (*HTZ78_01960*, DESeq2 *p* = 1.02 × 10^–72^), 35.6-fold increase in glucosylglycerol-phosphate synthase (*HTZ78_01970*, DESeq2 *p* = 2.56 × 10^–84^), and a 4.6-fold increase in glucosylglycerol 3-phosphatase (*HTZ78_06805*, DESeq2 *p* = 4.29 × 10^–25^) (Figure 4A). A previous metabolic profiling study also detected a significantly higher level (23.1-fold increase with Mann-Whitney test, *p* = 0.002) of glucosylglycerol in *Synechocystis* sp. PCC 7338 compared to *Synechocystis* sp. PCC 6803, which uses glucosylglycerol as the main compatible solute. Thus, our results support that *Synechocystis* sp. PCC 7338 uses glucosylglycerol as a compatible solute to respond to the osmotic stress. In addition, the ABC transporters with specific functions, such as transport of amino acids, branched-chain amino acids, and carbohydrates were highly expressed in *Synechocystis* sp. PCC 7338 (Supplementary Figure 4D). The highly expressed genes in *Synechocystis* sp. PCC 7338 also include aquaporin (*HTZ78_10655*) and sodium/proton antiporters (*HTZ78_01555*, *HTZ78_09010*, and *HTZ78_13585*), which are known to be involved in response to osmolarity oscillations and salt tolerance in cyanobacteria, respectively (Supplementary Data 4) (Waditee et al., 2002; Akai et al., 2012). The bacterial secretion system genes and motility genes encoding membrane proteins that utilize ATP to operate their functions were highly expressed in *Synechocystis* sp. PCC 7338, justifying their higher demand for ATP. Interestingly, comparing the regulatory regions of the highly expressed ABC transporters, aquaporin, antiporters, secretion system genes, and motility genes, most of them (15 out of 17) had degenerated (orphan) promoter regions (Supplementary Data 3).

Among the photosynthesis genes, those related to PSI, ATP synthase, and antenna proteins were upregulated in *Synechocystis* sp. PCC 7338 (Figure 4B). In addition, 50.82% of the genes related to porphyrin and chlorophyll metabolism (31 out of 61) were upregulated while 16.39% were downregulated (Figure 4C). On the contrary, genes related to PSII tend to be downregulated, especially the genes encoding D1 proteins which are responsible for the PSII repair. The low D1 protein synthesis rate can cause vulnerability to even low illumination and generating reactive oxygen species (ROS), which induces oxidative stress within the cell (Nishiyama et al., 2001; Allakhverdiev and Murata, 2004; Nishiyama et al., 2006). However, the expression of genes responding to oxidative stress decreased, indicating that the light energy transferred to PSII was insufficient to cause oxidative stress in cells (Supplementary Data 4). An antenna protein phycobilisome (PBS) mainly binds to PSII to transfer the light energy (Chang et al., 2015; Luimstra et al., 2019). However, the energy is transferred directly from PBS to PSI through state transition or PBS-PSI complex formation under certain circumstances, such as plastoquinone pool reduction and high ATP demand (Allen et al., 1981; Mullineaux and Allen, 1990). For example, in *Anabaena* sp., the cyclic electron transport through PSI provides ATP for nitrogen fixation under the light-limited conditions by forming a PBS-CpcL-tetrameric PSI supercomplex (Watanabe et al., 2014). CpcL is a variant of the rod-core linker CpcG, which possesses a hydrophobic region at its C-terminus. Similar to *Anabaena* sp., increased expression of PSI, ferredoxin-NADP (+) reductase (FNR), and ATP synthase genes were observed in *Synechocystis* sp. PCC 7338, indicating the activation of cyclic electron transport chain. Furthermore, genes encoding NAD(P)H-quinone oxidoreductase complex (NDH-1), which is involved in NDH-1-mediated cyclic electron transport, were also upregulated (Supplementary Table 6) (Laughlin et al., 2020). In addition, among the two *cpcG* in *Synechocystis* sp. PCC 7338, the one with a hydrophobic region in the C-terminus (*HTZ78_06190*; CpcL) was highly upregulated, suggesting the formation of a PBS-CpcL-PSI supercomplex in *Synechocystis* sp. PCC 7338 (Figure 4D). Independent validation of the RNA-seq data was performed through quantitative PCR by selection of genes that have a broad range of fold changes between *Synechocystis* sp. PCC 7338 and *Synechocystis* sp. PCC 6803. The comparison of the RNA-seq data and quantitative PCR results showed high R^2^ value (0.97), indicating the reliability of the quantification based on RNA-seq data (Supplementary Figure 4E). In summary, *Synechocystis* sp. PCC 7338 may have evolved by increased distribution of energy to PSI than PSII, thus allowing it to adapt to situations with high ATP demand and insufficient light (Figure 4E). This interpretation agrees with the previous observations of increased PSI/PSII ratio under high salt stress, and consistent with the suggestion that optimal photosynthesis can be achieved with short-term state transition and long-term PSI-specific complex formation in cyanobacteria (Hagemann, 2011; Watanabe et al., 2014).

## Discussion

We obtained a high-quality genome sequence of marine cyanobacteria *Synechocystis* sp. PCC 7338 and aligned the same with multi-omics data for systematic analysis of its survival under harsh environmental conditions of high salinity and high osmotic pressure. Compared to freshwater *Synechocystis* sp. PCC 6803, *Synechocystis* sp. PCC 7338 is expected to actively use the cyclic electron transport through PSI to replenish the insufficient ATP availability under the conditions (Figure 4E). It has been suggested that ATP demand increases under salt stress, which might reduce the carbon fixation rate (van Thor et al., 2000). However, in *Synechocystis* sp. PCC 7338, the gene expression related to carbon fixation increased slightly, indicating no significant reduction in carbon fixation rate (Figure 4E and Supplementary Figure 5).

A significant downregulation of the D1 protein gene was observed in *Synechocystis* sp. PCC 7338, which agrees with the previous results that the transcription and subsequent production D1 protein is inhibited by salt stress (Figure 4B; Allakhverdiev and Murata, 2008; Yang et al., 2020). As a result, the repair rate of damaged PSII would slow down, and oxidative stress would occur when excessive energy is transferred to PSII. Thus, adaptation toward distributing electrons to PSI rather than PSII may be beneficial for survival of marine cyanobacteria. Interestingly, the comparison of 5′-UTR length of orthologs between the two strains showed that the downregulated D1 encoding genes have a much shorter 5′-UTR in *Synechocystis* sp. PCC 7338 (Figure 3C and Supplementary Figure 3B). Considering the regulation by cis- or trans-regulatory elements in the longer 5′-UTR, it is possible that the reduction in the 5′-UTR length of D1 encoding genes was a strategic change to avoid negative regulation from salt stress or oxidative stress. When the regulatory regions of the orthologs were compared, the promoter regions of PSII genes were more divergent than those of PSI genes, suggesting that the different regulatory mechanism of PSII to respond more sensitively to stress such as excessive light or oxidative stress in the two strains (Figure 3D). In addition to the photosynthesis-related genes, the degenerated promoter regions of the genes involved in response to stress conditions, such as aquaporin and sodium/proton antiporters, inferred the diverged regulatory mechanisms in the two strains. Taken together, our analysis suggests that *Synechocystis* sp. PCC 7338 adapted to a different homeostatic balance compared to the freshwater *Synechocystis* sp. PCC 6803, by evolving its photosynthetic mechanism and different regulatory network through changes in regulatory sequences to avoid various stresses, such as high salinity, high osmotic pressure, and oxidative stress in the marine environment.

## Data Availability Statement

The datasets presented in this study can be found in online repositories. The names of the repository/repositories and accession number(s) can be found below: https://www.ncbi.nlm.nih.gov/, PRJNA629670.

## Author Contributions

SC and B-KC designed and supervised the project. YJ, S-JH, and S-HC performed experiments. YJ, S-JH, S-HC, SC, and B-KC analyzed the data. YJ, HL, H-KC, D-MK, C-GL, SC, and B-KC wrote the manuscript. All authors have read and approved the final manuscript.

## Conflict of Interest

The authors declare that the research was conducted in the absence of any commercial or financial relationships that could be construed as a potential conflict of interest.

## Footnotes

1 https://www.ncbi.nlm.nih.gov/sra/PRJNA629670
