# Supplementary material for: Multi-Omic Analyses Reveal Habitat Adaptation of Marine Cyanobacterium Synechocystis sp. PCC 7338
Source: Front Microbiol. 2021 May 13;12:667450. doi: 10.3389/fmicb.2021.667450 (PMC8155712; doi:10.3389/fmicb.2021.667450)
Supplement: Supplementary file 1 [file Data_Sheet_1.docx]

Supplementary Material

**This PDF file includes:**

Supplementary Figures S1 to S5

Supplementary Tables S1 to S6

References

**Other supplementary materials for this manuscript include the following:**

Supplementary Data S1 to S4

# Supplementary Figures


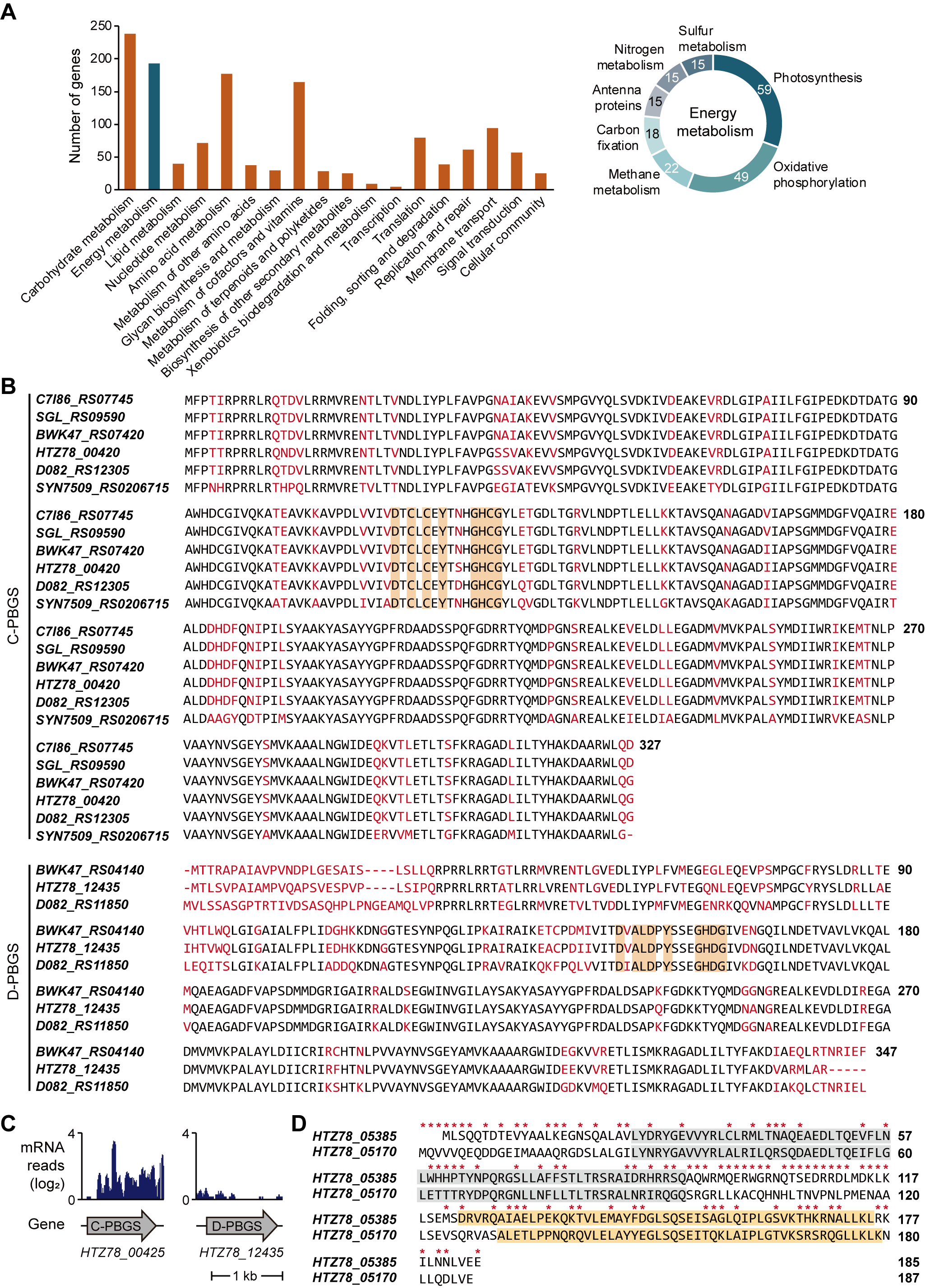


Supplementary Figure S1. Comparison of the genes in *Synechocystis* sp. PCC 7338 with other *Synechocystis*. (A) The Kyoto Encyclopedia of Genes and Genomes (KEGG) pathway analysis of annotated coding sequences of *Synechocystis* sp. PCC 7338. (B) The amino acid sequences of C-PBGS in *Synechocystis* sp. IPPAS B-1465 (C7I86_RS07745), *Synechocystis* sp. PCC 6803 (SGL_RS09590), *Synechocystis* sp. CACIAM 05 (BWK47_RS07420), *Synechocystis* sp. PCC 7338 (HTZ78_00420), *Synechocystis* sp. PCC 6714 (D082_RS12305), and *Synechocystis* sp. PCC 7509 (SYN7509_RS0206715), and D-PBGS in *Synechocystis* sp. CACIAM 05 (BWK47_RS04140), *Synechocystis* sp. PCC 7338 (HTZ78_12435), and *Synechocystis* sp. PCC 6714 (D082_RS11850). Orange boxes indicate the active sites and red characters indicate the varying sequences. (C) mRNA read profiles of C-PBGS (HTZ78_00425) and D-PBGS (HTZ78_12435). (D) Comparison of extra sigma factor (HTZ78_05385) and SigI (HTZ78_05170) sequences. Red asterisks indicate the varying sequences. Grey boxes indicate sigma-70 region 2 and orange boxes indicate sigma-70 region 4.


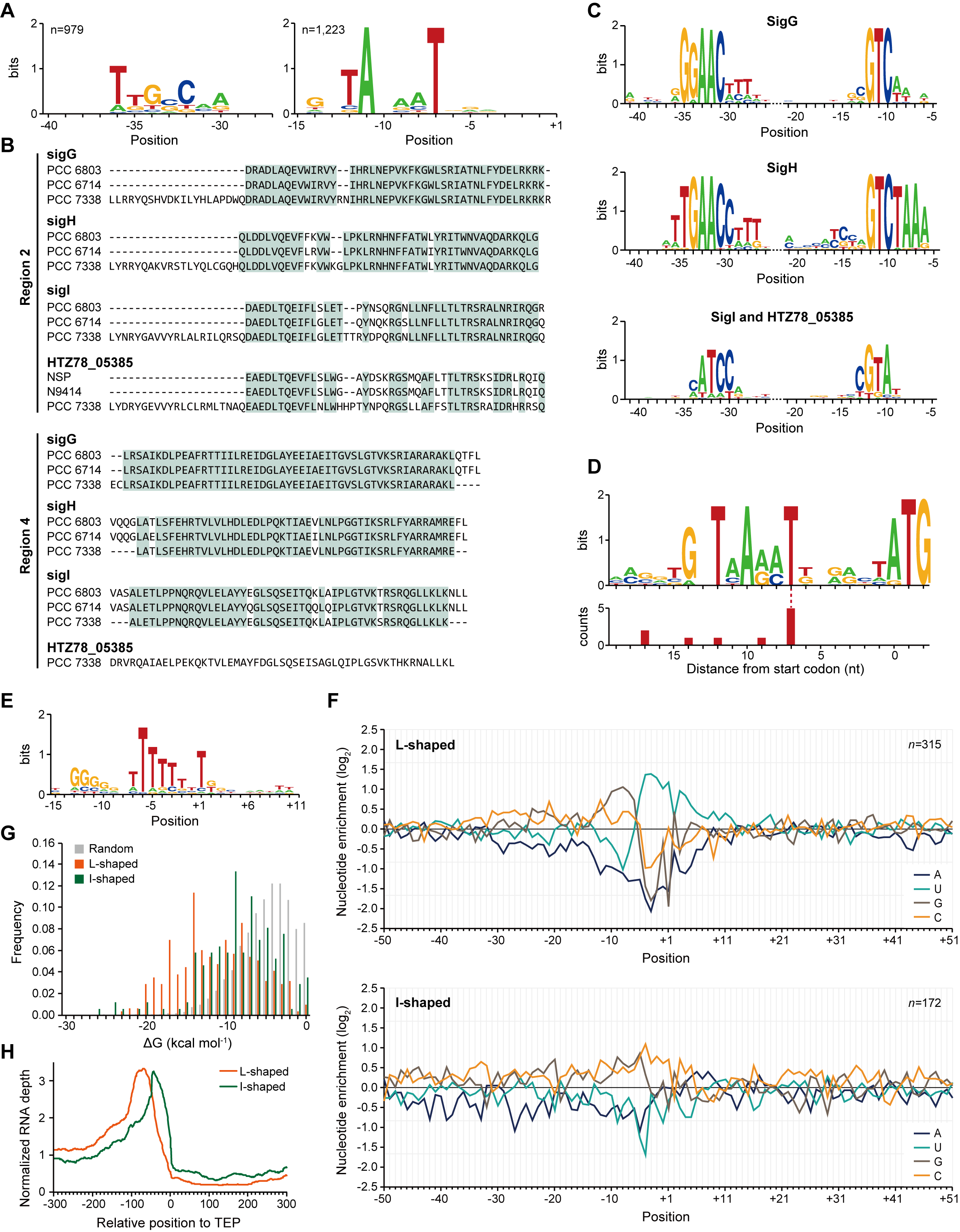


Supplementary Figure S2. The regulatory elements revealed by transcription start sites (TSSs) and transcript 3′-end positions (TEPs) identification. (A) Conserved promoter sequences detected in the upstream sequences of previously identified TSSs in *Synechocystis* sp. PCC 6803 (Mitschke et al., 2011;Kopf et al., 2014). (B) The aligned region 2 and region 4 sequences of SigG, SigH, SigI, and HTZ78_05385 of *Synechocystis* sp. PCC 7338 with the closest sequences of *Synechocystis* sp. PCC 6803 (PCC 6803) and *Synechocystis* sp. PCC 6714 (PCC 6714), or *Nodularia spumigena* CENA 596 (NSP) and *N. spumigena* CCY 9414 (N9414). The region 2 and region 4 of sigma factors of *Synechocystis* sp. PCC 7338 were obtained from Pfam sequence search, and others were obtained from previous study (El-Gebali et al., 2019;Todor et al., 2020). The sequences were aligned by using ClustalW method, and the closest sequences were determined by using Maximum likelihood phylogenetic analysis (Kumar et al., 2018). Cyan boxes indicate the conserved sequences. (C) The predicted promoter motifs of the ECF sigma factor clusters containing SigG, or SigH, or SigI and HTZ78_05385. The lists of the ECF sigma factor clusters and the predicted promoter motifs were obtained from previous study (Todor et al., 2020). (D) The conserved sequence search in the upstream region of start codon of leaderless transcripts using MEME. (E) Conserved motif sequences detected in TEPs in *Synechocystis* sp. PCC 6803. (F-H) Features of L- and I-shaped TEPs. (F) Nucleotide enrichment calculated in ±50 nt sequences from L-shaped TEPs (top) and I-shaped TEPs (bottom). The ratio of each nucleotide at each position was normalized with those of randomly selected intergenic positions (n=10,000). (G) The folding energy was calculated at upstream sequences of L-shaped TEPs, I-shaped TEPs, or at randomly selected intergenic positions (n=10,000). (H) RNA expression profiles near the L-shaped TEPs and I-shaped TEPs.


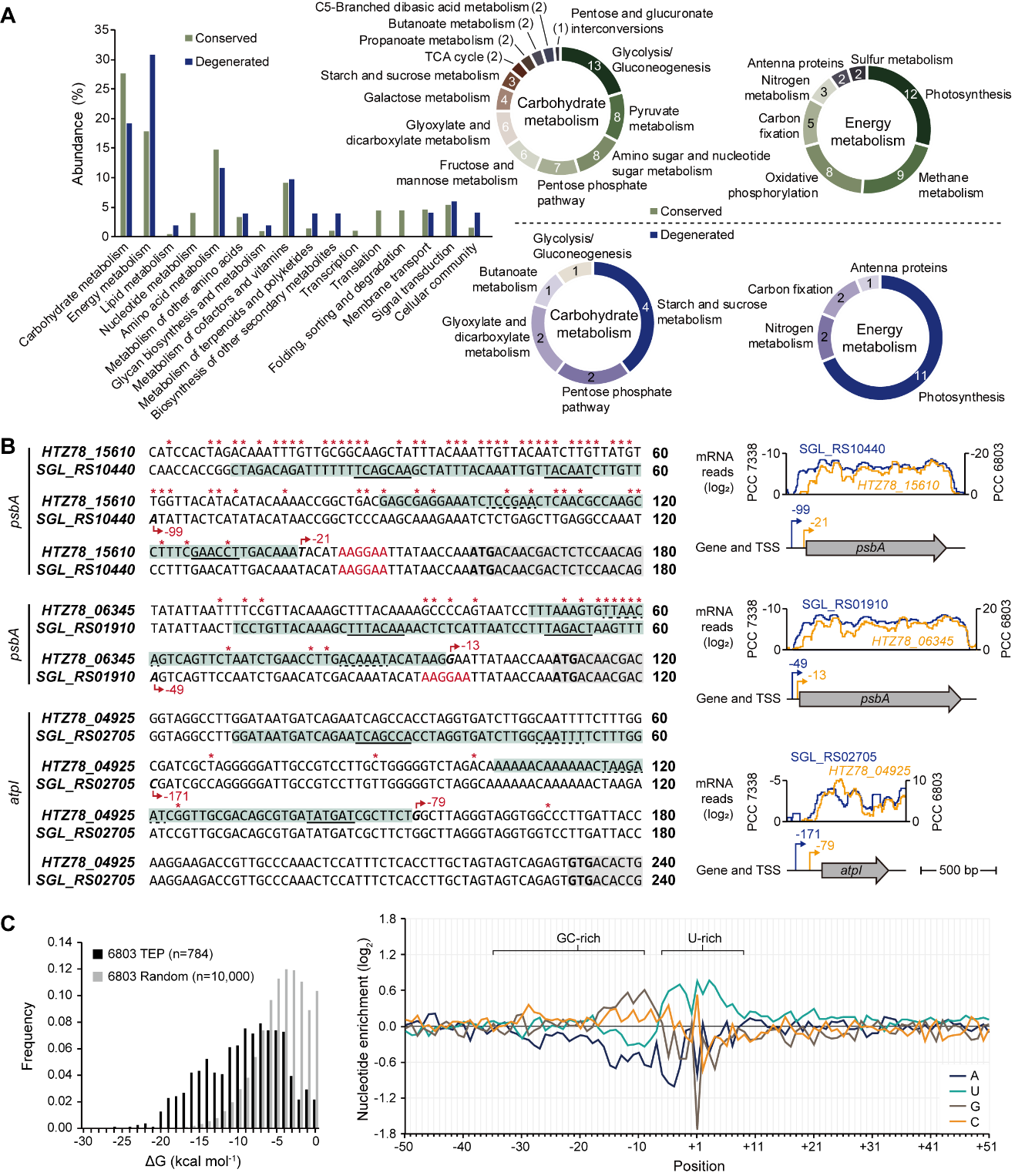


Supplementary Figure S3. Comparison of regulatory elements with *Synechocystis* sp. PCC 6803. (A) The KEGG pathway analysis of orthologs having conserved 5′-UTR length or degenerated 5′-UTR length. (B) The examples of orthologs having different length of 5′-UTRs. Grey boxes indicate the coding sequences and cyan boxes indicate the orphan promoter regions. Red asterisks indicate the varying sequences. Red arrows indicate the TSSs, and the relative positions of the TSSs to the start codons are designated near the red arrows. The promoter sequences detected from MEME search are underlined with solid lines, and the predicted promoter sequences are underlined with dotted lines. The RBS sequences are indicated as red characters, and the start codons are indicated as bold characters. Right panel shows the RNA expression profiles of each example. (C) The left panel shows the folding energy distribution calculated at upstream sequences from TEPs of *Synechocystis* sp. PCC 6803 or at randomly selected intergenic positions (n = 10,000). The right panel shows nucleotide enrichment calculated in ± 50 nt sequences from TEPs of *Synechocystis* sp. PCC 6803. The ratio of each nucleotide at each position was normalized with those of randomly selected intergenic positions.


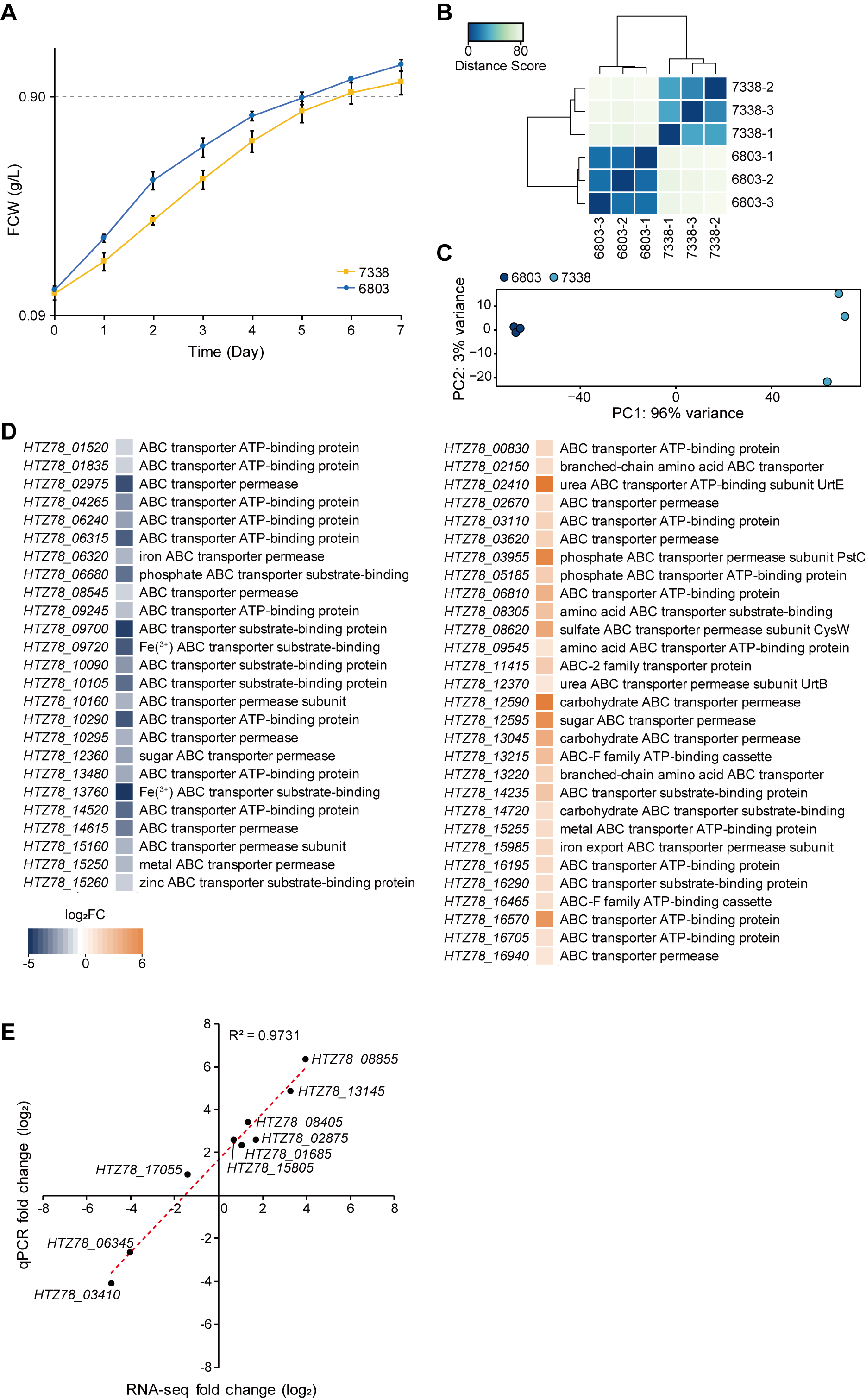


Supplementary Figure S4. Confirmation of RNA-seq data reproducibility and analysis of the differentially expressed genes (DEGs). (A) Growth curves of *Synechocystis* sp. PCC 7338 and *Synechocystis* sp. PCC 6803. The sampling point was indicated as dotted line. The y-axis is in log scale. (B, C) The distance between samples were represented as a heatmap (B) or a PCA plot (C). (D) Log_2_ fold changes of DEGs encoding ABC transporters. (E) Comparison between fold changes from RNA-seq and qPCR data. HTZ78_07965 (*sigA*) was used as a control gene.


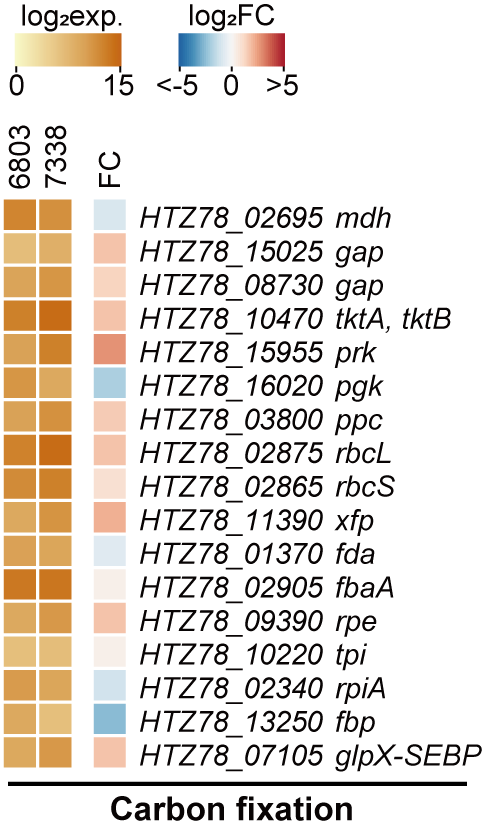


Supplementary Figure S5. Log_2_ mRNA expression levels and log_2_ fold changes of orthologs related to carbon fixation in *Synechocystis* sp. PCC 7338 and *Synechocystis* sp. PCC 6803.

# Supplementary Tables

Supplementary Table S1. Primers used in this study

| Gene | Template strand | Sequence (5'→3') | Product length |
| --- | --- | --- | --- |
| Primers for *Synechocystis* sp. PCC 7338 | | | |
| HTZ78_03410 | Plus | TGCGGATCCGGAAATCATTC | 101 |
|  | Minus | CGTAGTTAGCCTGGGACTTC |  |
| HTZ78_06345 | Plus | GTCGGTTGGTTCGGTACTC | 107 |
|  | Minus | TCACGGATACCGTCGATGT |  |
| HTZ78_17055 | Plus | GCTAAAGCGTGATCGTTTCG | 110 |
|  | Minus | AGGAAGTAACGAAGGTGGTG |  |
| HTZ78_15805 | Plus | CTGCGGAAGAAGCTGGTCTA | 100 |
|  | Minus | TCGGATTGGTCAACACTACC |  |
| HTZ78_01685 | Plus | CCGTAGGCGAAGGTCTAAAG | 104 |
|  | Minus | GGGCTGTTCGGCAAATAAAG |  |
| HTZ78_08405 | Plus | CTGGTTTAATTTCGCCCCGA | 100 |
|  | Minus | GAGGTAGTTCTGGGACTTAGC |  |
| HTZ78_02875 | Plus | TCGCCTGACCTACTATACCC | 100 |
|  | Minus | GGCTTCTTCAGCAGGTACAC |  |
| HTZ78_13145 | Plus | CCGGGTGAACTGGATCGTAT | 100 |
|  | Minus | GTTTAACGATGGTTTCGCGG |  |
| HTZ78_08855 | Plus | TTGGTTGTACCCAATGCGTC | 107 |
|  | Minus | ATCCTCGGTTCTAGGGGAAG |  |
| HTZ78_07965 | Plus | GCTGAATCCCCTGAACTAGAC | 110 |
|  | Minus | GCGGCAACTTCCTGTTCTAA |  |
| Primers for *Synechocystis* sp. PCC 6803 | | | |
| SGL_RS15155 | Plus | TGCGGATCCGGAAATCATTC | 101 |
|  | Minus | CGTAGTTGGCCTGGGATTTT |  |
| SGL_RS01910 | Plus | GGTTGGTTCGGTACCTTGAT | 102 |
|  | Minus | ACGGATACCGTCGATGTCAA |  |
| SGL_RS16825 | Plus | GCTAAAGCGTGATCGTTTCG | 110 |
|  | Minus | AGGAAGTAACGAAGGTGGTG |  |
| SGL_RS13450 | Plus | CTATATCCTCGATGCTGCGG | 102 |
|  | Minus | ACACTACCAGCGGTGATCTT |  |
| SGL_RS05250 | Plus | TTAGATGCTTTGAGCGCTACC | 104 |
|  | Minus | GAGCAGCGTTGGAAACGATA |  |
| SGL_RS09810 | Plus | AATTTCGCTCCCTCAGCTTC | 106 |
|  | Minus | CGTTGGTGGTATTGAGGAAGT |  |
| SGL_RS13415 | Plus | TCGCCTGACCTACTATACCC | 100 |
|  | Minus | GGCTTCTTCAGCAGGTACAC |  |
| SGL_RS08580 | Plus | GCGAACTGGATCGTATTAAAGC | 104 |
|  | Minus | GCCAGCTTGTTTAACGATGG |  |
| SGL_RS12620 | Plus | TTGGTTGTACCCAATGCGTC | 108 |
|  | Minus | AATCCTCGGTTCTAGGGGAG |  |
| SGL_RS17245 | Plus | GCCGAACTGGAGCAAGAAA | 109 |
|  | Minus | CTTCTGTAGCGGCGACTTC |  |

Supplementary Table S2. Genome characteristics of genome sequenced *Synechocystis* sp. strains

|  | **PCC 7338** | **PCC 6803** | **PCC 6714** | **CACIAM 05** | **IPPAS B-1465** | **PCC 7509** |
| --- | --- | --- | --- | --- | --- | --- |
| Genome size (Mbp) | 3.70 | 3.57 | 3.49 | 3.58 | 3.57 | 4.91 |
| Total genes | 3589 | 3699 | 3499 | 3351 | 3693 | 4894 |
| Total CDS | 3385 | 3559 | 3312 | 3168 | 3546 | 4608 |
| tRNA | 42 | 41 | 42 | 42 | 41 | 50 |
| rRNA | 6 | 6 | 6 | 3 | 6 | 6 |
| other RNA | 4 | 4 | 4 | 4 | 4 | 4 |
| Pseudogenes | 152 | 89 | 135 | 134 | 96 | 226 |
| GC content of genome (%) | 47.82 | 47.70 | 47.80 | 47.80 | 47.70 | 41.70 |
| Number of plasmids | 3 | 4 | 3 | 0 | 6 | 0 |

Supplementary Table S3. The numbers of core, dispensable, and specific genes obtained by pan-genome analysis

|  | **PCC 7338** | **PCC 6803** | **PCC 6714** | **CACIAM 05** | **IPPAS B-1465** | **PCC 7509** |
| --- | --- | --- | --- | --- | --- | --- |
| Core | 1,441 | 1,442 | 1,425 | 1,431 | 1,442 | 1,457 |
| Dispensable | 1,544 | 2,066 | 1,504 | 1,538 | 2,073 | 186 |
| Specific | 396 | 48 | 383 | 185 | 31 | 2,963 |

Supplementary Table S4. Additional genes in *Synechocystis* sp. PCC 7338 compared to other freshwater *Synechocystis* sp. strains

| **Gene ID** | **KEGG ID** | **Function** | **Length (amino acid)** |
| --- | --- | --- | --- |
| **Related to environment** | | | |
| HTZ78_10130 | - | OsmC family protein | 155 |
| HTZ78_01195 | - | mechanosensitive ion channel | 307 |
| HTZ78_07470 | - | mechanosensitive ion channel | 551 |
| HTZ78_08175 | K05802 | mechanosensitive ion channel | 441 |
| HTZ78_10625 | K16052 | mechanosensitive ion channel | 607 |
| HTZ78_10630 | K16052 | mechanosensitive ion channel | 370 |
| HTZ78_15990 | K03442 | mechanosensitive ion channel family protein | 437 |
| **Related to sigma factor** | | | |
| HTZ78_05385 | K03088 | sigma-70 family RNA polymerase sigma factor | 185 |
| HTZ78_05390 | - | anti-sigma factor | 253 |

Supplementary Table S5. The genes having Fur binding motif in their 500 nt upstream sequences from the start codons (searched by MEME) or 100 nt upstream sequences from TSSs (searched by FIMO).

| **MEME results** | | | |
| --- | --- | --- | --- |
| **Gene** | **Position from start codon** | **p-value** | **Motif** |
| HTZ78_13180 | 479 | 6.89E-09 | AAAAAATTGTTAGTTTTTTT |
| HTZ78_10020 | 106 | 2.63E-07 | CAAAAATTTGTGGTTTATTT |
| HTZ78_05040 | 404 | 2.63E-07 | CAAAATTTTTTTTCCCTTTC |
| HTZ78_14380 | 463 | 3.45E-07 | AAAAAATTTATATTCTTTTA |
| HTZ78_03360 | 377 | 3.45E-07 | CCAAAATTAATAACCTATTT |
| HTZ78_14195 | 477 | 4.47E-07 | GAAAATTTAGTATTTCTTGA |
| HTZ78_13925 | 477 | 4.47E-07 | GAAAATTTAGTATTTCTTGA |
| HTZ78_11305 | 26 | 4.47E-07 | CAAAAACTGTTAGCTTGTTT |
| HTZ78_06220 | 64 | 4.47E-07 | CAAAAATTGATAATTTTTAT |
| HTZ78_00225 | 477 | 4.47E-07 | GAAAATTTAGTATTTCTTGA |
| HTZ78_01235 | 82 | 8.30E-07 | TAAAATTTCTTAACTTTTGT |
| HTZ78_14040 | 120 | 1.05E-06 | CCAACTTTTGTAGCTTTTTC |
| HTZ78_07690 | 99 | 1.05E-06 | AAAAAGTTGATAGTTCATGT |
| HTZ78_09775 | 39 | 1.18E-06 | CAAAAATTAACATTTCTTGG |
| HTZ78_03510 | 210 | 1.33E-06 | AAAAATTTTGTAATTGTTTA |
| HTZ78_01510 | 455 | 1.66E-06 | CAAACTTTAATTTTTTTGGT |
| HTZ78_05400 | 432 | 1.85E-06 | CCAAAATTAATAATTATCTT |
| HTZ78_04390 | 392 | 1.85E-06 | TAAAAATTGTTTTTTGTTGT |
| HTZ78_16685 | 471 | 2.07E-06 | CCAAAATTATTCTCTTTGTT |
| HTZ78_04600 | 382 | 2.07E-06 | GCAAATTTTTTTACTGTTTT |
| HTZ78_12905 | 227 | 2.30E-06 | TAAGATTTTATAATTCTTTT |
| HTZ78_03355 | 444 | 2.30E-06 | CAAAGATTTATAGATTTTTT |
| HTZ78_09535 | 358 | 2.84E-06 | CAAAAATTCATAATCAGTGT |
| HTZ78_07310 | 340 | 2.84E-06 | ACAAAATTAATACTCTTTGT |
| HTZ78_07080 | 125 | 2.84E-06 | CCACATTTTTTAGATATTTT |
| HTZ78_14790 | 323 | 3.15E-06 | CCAAAGTTACTATTTTTTGC |
| HTZ78_05820 | 453 | 3.86E-06 | CAAAATTTTTCAGCTTAGGT |
| HTZ78_09990 | 274 | 4.71E-06 | CAAAAACTTAAATCTTTTGC |
| HTZ78_02465 | 21 | 5.19E-06 | CAAAATTAGCTTACTATTTT |
| HTZ78_08765 | 172 | 5.72E-06 | CAAAATCTAGCTTTTTTTCT |
| HTZ78_02415 | 402 | 6.29E-06 | CAAAAATTCTTGGCTGTTTG |
| HTZ78_00020 | 403 | 6.29E-06 | GCAAAGTTAACAATTATTTT |
| HTZ78_03940 | 449 | 7.59E-06 | GAAAAATAAATATCTAATTT |
| HTZ78_03385 | 194 | 7.59E-06 | TAAAAATTGATAAATCCTTT |
| HTZ78_15990 | 188 | 8.32E-06 | GAAAAACAGTTAATTTTTTT |
| HTZ78_08965 | 239 | 8.32E-06 | CAACAATTTGAGATTTTTTT |
| HTZ78_02120 | 271 | 8.32E-06 | CAAAATTTAATAATCCCTCA |
| HTZ78_00345 | 386 | 8.32E-06 | CAAAATTTGGTCGCACTGTT |
| HTZ78_01190 | 184 | 9.12E-06 | AAAACTTTATCTTTTCTTTT |
| HTZ78_05310 | 401 | 1.19E-05 | CGAAATTTTATTGCCTTTGA |
| HTZ78_08585 | 160 | 1.30E-05 | AAAAATCTGGCTTCTATTGT |
| HTZ78_04290 | 478 | 1.82E-05 | CAAAAGTTGTTGCTCTTTTA |
| HTZ78_07695 | 320 | 2.33E-05 | TAGAAATTTATAGTTTTTTG |
| **FIMO results** | | | |
| **Gene** | **position from TSS** | **p-value** | **Motif** |
| HTZ78_00120 | 48 | 3.39E-07 | AAAAAGTTGCTAATTTTTTT |
| HTZ78_00190 | 77 | 5.03E-05 | CCAAAATTCCCTATCATTTA |
| HTZ78_00325 | 33 | 9.44E-09 | TAAAAATTAATATTTTTTTT |
| HTZ78_00985 | 69 | 2.82E-06 | GCAAAATTCTCTGTTTTTTT |
| HTZ78_01075 | 35 | 1.52E-05 | CCAACGTTAGTTGCTTCTTT |
| HTZ78_01135 | 57 | 2.42E-05 | AAAGATTTGCTCGCTTTTTA |
| HTZ78_02660 | 7 | 3.66E-05 | CAAAAATTAATTTCCGGGGA |
| HTZ78_05555 | 24 | 7.06E-05 | TCAAATTTTAAATCTAATGC |
| HTZ78_06050 | 37 | 1.06E-05 | GAAGAATTGTTGACTTTTTC |
| HTZ78_06085 | 3 | 5.30E-05 | GAAGCATTAATGATCATTTT |
| HTZ78_06170 | 37 | 7.14E-05 | TGAAAATTAGCTTTTTTTTA |
| HTZ78_06255 | 4 | 8.16E-05 | GCAAAGCTTATTGTCTATCT |
| HTZ78_06620 | 33 | 4.15E-07 | AAAAAATTGATTCCCTTTTT |
| HTZ78_06655 | 12 | 1.98E-06 | GCAAAATTTTTTGCCCTTTC |
| HTZ78_07050 | 25 | 1.49E-05 | ACAAAATTTTTCGCCAATTT |
| HTZ78_07765 | 8 | 5.27E-05 | GAAAATTTTCTTTTTTACTA |
| HTZ78_08510 | 52 | 6.34E-06 | CAACATTTATTGTTTAATGT |
| HTZ78_08865 | 44 | 8.42E-06 | AAAAAATTAATCTTTTCTGC |
| HTZ78_09360 | 63 | 3.49E-05 | CAAACTTTTTTTGTCACTTG |
| HTZ78_09550 | 37 | 3.13E-08 | TAAAATTTTGTATCTTTTTT |
| HTZ78_10445 | 35 | 1.72E-05 | AAACAATTTGTTGCTGTTGG |
| HTZ78_10545 | 28 | 8.29E-05 | TAGAAATTTTAATTTTTTTA |
| HTZ78_11430 | 26 | 5.89E-06 | GAAACTTTGTTAATTTTTGG |
| HTZ78_11675 | 38 | 1.37E-05 | CAAAAGTATGCTGTTTTTGT |
| HTZ78_12890 | 54 | 2.91E-05 | CGAAAACTTATTCCCTTTTT |
| HTZ78_13060 | 81 | 4.46E-05 | TAAAAATTTATGACTAATCC |
| HTZ78_13160 | 44 | 3.53E-05 | AAAAATTTTCTGTCCAATTC |
| HTZ78_13375 | 71 | 5.86E-05 | CAAACTTTAATGGCAGTTGC |
| HTZ78_14555 | 31 | 4.76E-05 | AAAAATTATGATTTTTTGTT |
| HTZ78_15055 | 59 | 9.49E-06 | CCAAATTTAGTGTTTTCGTT |
| HTZ78_15330 | 7 | 8.65E-05 | CGAAATTTGGAACCCTTTTC |
| HTZ78_15580 | 44 | 3.47E-05 | CAAAGATTAACAATTTAGTT |
| HTZ78_16245 | 19 | 9.12E-05 | CAAAAATTAACATTTTTATC |
| HTZ78_16580 | 69 | 6.95E-05 | TAAAATTTTGCTTTTTAGCT |

Supplementary Table S6. RNA-seq fold changes of NAD(P)H-quinone oxidoreductase complex (NDH-1) and ferredoxin-NADP (+) reductase (FNR) genes between *Synechocystis* sp. PCC 7338 and *Synechocystis* sp. PCC 6803

| Gene | log2  FC | adj.  P-val | Function | KO ID |
| --- | --- | --- | --- | --- |
| HTZ78_10390 | 1.64 | 7.6E-08 | ndhA; NAD(P)H-quinone oxidoreductase subunit 1 | K05572 |
| HTZ78_04760 | 0.88 | 3.2E-04 | ndhB; NAD(P)H-quinone oxidoreductase subunit 2 | K05573 |
| HTZ78_12120 | -0.17 | 5.5E-01 | ndhC; NAD(P)H-quinone oxidoreductase subunit 3 | K05574 |
| HTZ78_13780 | -6.52 | 1.4E-131 | ndhD; NAD(P)H-quinone oxidoreductase subunit 4 | K05575 |
| HTZ78_08865 | 0.33 | 1.5E-01 | ndhD; NAD(P)H-quinone oxidoreductase subunit 4 | K05575 |
| HTZ78_12645 | 1.88 | 1.6E-09 | ndhD; NAD(P)H-quinone oxidoreductase subunit 4 | K05575 |
| HTZ78_12875 | -0.05 | 8.9E-01 | ndhD; NAD(P)H-quinone oxidoreductase subunit 4 | K05575 |
| HTZ78_10405 | 2.37 | 1.1E-07 | ndhE; NAD(P)H-quinone oxidoreductase subunit 4L | K05576 |
| HTZ78_12880 | 0.11 | 7.8E-01 | ndhF; NAD(P)H-quinone oxidoreductase subunit 5 | K05577 |
| HTZ78_03310 | 0.15 | 5.6E-01 | ndhF; NAD(P)H-quinone oxidoreductase subunit 5 | K05577 |
| HTZ78_12640 | 1.02 | 2.7E-03 | ndhF; NAD(P)H-quinone oxidoreductase subunit 5 | K05577 |
| HTZ78_10400 | 3.04 | NA | ndhG; NAD(P)H-quinone oxidoreductase subunit 6 | K05578 |
| HTZ78_06610 | 1.09 | 2.4E-04 | ndhH; NAD(P)H-quinone oxidoreductase subunit H | K05579 |
| HTZ78_10395 | 2.50 | 1.0E-09 | ndhI; NAD(P)H-quinone oxidoreductase subunit I | K05580 |
| HTZ78_12110 | 1.87 | 1.0E-08 | ndhJ; NAD(P)H-quinone oxidoreductase subunit J | K05581 |
| HTZ78_12115 | 1.49 | 2.2E-08 | ndhK; NAD(P)H-quinone oxidoreductase subunit K | K05582 |
| HTZ78_13310 | 0.23 | 4.9E-01 | ndhL; NAD(P)H-quinone oxidoreductase subunit L | K05583 |
| HTZ78_15825 | 1.20 | 2.9E-04 | ndhM; NAD(P)H-quinone oxidoreductase subunit M | K05584 |
| HTZ78_09350 | -0.06 | 8.4E-01 | ndhN; NAD(P)H-quinone oxidoreductase subunit N | K05585 |
| HTZ78_15960 | 1.40 | 4.2E-04 | petH; ferredoxin--NADP+ reductase | K02641 |

# References

El-Gebali, S., Mistry, J., Bateman, A., Eddy, S.R., Luciani, A., Potter, S.C., et al. (2019). The Pfam protein families database in 2019. *Nucleic Acids Res.* 47**,** D427-D432.

Kopf, M., Klahn, S., Scholz, I., Matthiessen, J.K., Hess, W.R., and Voss, B. (2014). Comparative analysis of the primary transcriptome of *Synechocystis* sp. PCC 6803. *DNA Res.* 21**,** 527-539.

Kumar, S., Stecher, G., Li, M., Knyaz, C., and Tamura, K. (2018). MEGA X: Molecular evolutionary genetics analysis across computing platforms. *Mol. Biol. Evol.* 35**,** 1547-1549.

Mitschke, J., Georg, J., Scholz, I., Sharma, C.M., Dienst, D., Bantscheff, J., et al. (2011). An experimentally anchored map of transcriptional start sites in the model cyanobacterium *Synechocystis* sp. PCC6803. *Proc. Natl. Acad. Sci. U. S. A.* 108**,** 2124-2129.

Todor, H., Osadnik, H., Campbell, E.A., Myers, K.S., Li, H., Donohue, T.J., et al. (2020). Rewiring the specificity of extracytoplasmic function sigma factors. *Proc. Natl. Acad. Sci. U. S. A.* 117**,** 33496-33506.
